# Crown-ether-programmed covalent organic framework nanochannels enable quasi-single-ion conductivity for solid-state lithium metal batteries

**DOI:** 10.1093/nsr/nwag098

**Published:** 2026-02-13

**Authors:** Yihang Nie, Shibin Li, Tingzhou Yang, Longjie He, Guo Feng, Yiting Shao, Qingying Li, Jiawei He, Mingliang Jin, Dan Luo, Xin Wang, Zhongwei Chen

**Affiliations:** Guangdong Provincial Key Laboratory of Optical Information Materials and Technology, South China Academy of Advanced Optoelectronics, International Academy of Optoelectronics at Zhaoqing, South China Normal University, Guangzhou 510006, China; Institute of Carbon Neutrality, Zhejiang Wanli University, Ningbo 315100, China; School of Chemistry and Chemical Engineering, Nantong University, Nantong 226019, China; Institute of Carbon Neutrality, Zhejiang Wanli University, Ningbo 315100, China; Guangdong Provincial Key Laboratory of Optical Information Materials and Technology, South China Academy of Advanced Optoelectronics, International Academy of Optoelectronics at Zhaoqing, South China Normal University, Guangzhou 510006, China; Guangdong Provincial Key Laboratory of Optical Information Materials and Technology, South China Academy of Advanced Optoelectronics, International Academy of Optoelectronics at Zhaoqing, South China Normal University, Guangzhou 510006, China; Guangdong Provincial Key Laboratory of Optical Information Materials and Technology, South China Academy of Advanced Optoelectronics, International Academy of Optoelectronics at Zhaoqing, South China Normal University, Guangzhou 510006, China; Guangdong Provincial Key Laboratory of Optical Information Materials and Technology, South China Academy of Advanced Optoelectronics, International Academy of Optoelectronics at Zhaoqing, South China Normal University, Guangzhou 510006, China; Guangdong Provincial Key Laboratory of Optical Information Materials and Technology, South China Academy of Advanced Optoelectronics, International Academy of Optoelectronics at Zhaoqing, South China Normal University, Guangzhou 510006, China; State Key Laboratory of Catalysis, Dalian Institute of Chemical Physics, Chinese Academy of Sciences, Dalian 116023, China; Institute of Carbon Neutrality, Zhejiang Wanli University, Ningbo 315100, China; Ningbo Key Laboratory of High Energy Density Battery, Yuyao Innovation Institute, Zhejiang Wanli University, Ningbo 315100, China; State Key Laboratory of Catalysis, Dalian Institute of Chemical Physics, Chinese Academy of Sciences, Dalian 116023, China

**Keywords:** lithium metal battery, solid-state electrolyte, covalent organic framework, single-ion conducting, migration mechanism

## Abstract

Solid-state lithium (Li) metal batteries are hindered by sluggish Li^+^ transport and anion-driven interfacial instabilities in polymer electrolytes. Herein, we develop a quasi-single-ion-conducting polymer electrolyte by embedding a crown ether-functionalized covalent organic framework (COF) into a fluorinated polymer matrix. Imine (C=N) linkages in the COF and polar fluorinated polymer domains cooperatively immobilize TFSI^−^ via electrostatic adsorption and pore-defined confinement, while the imine sites and crown ether oxygens dynamically decouple Li^+^ from its counter-anion and provide exchangeable coordination nodes for rapid interlayer migration along ordered COF channels. As a result, the electrolyte delivers a high ionic conductivity of 1.15 × 10^−3^ S cm^−1^ with a high Li^+^ transference number of 0.91, establishing a continuous Li^+^-preferential transport network that homogenizes ion flux, promotes the formation of thin and compact interphases, and stabilizes Li anodes and high-voltage cathodes. This crown ether–COF design establishes a broadly applicable design paradigm for decoupling ion transport and interfacial chemistry, paving the way toward next-generation long-lifetime Li metal batteries.

## Teaser

Crown-ether COF within a fluorinated polymer creates an anion-bypass, O-belt Li^+^ hopping network, enabling quasi-single-ion conduction and long-life solid-state Li metal batteries.

## INTRODUCTION

Solid-state lithium metal batteries (SSLMBs) are widely regarded as next-generation energy storage systems due to their potential to combine high energy density with intrinsic safety [1–4]. Replacing flammable liquid electrolytes with solid polymer electrolytes can improve mechanical flexibility and eliminate the risk of leakage, but a central bottleneck of conventional solid polymer electrolyte lies in the facile migration of anions [5–8]. Because lithium (Li) ion transport is coupled to sluggish polymer segmental dynamics, the majority of ionic current is instead carried by counter-anions, leading to low Li^+^ transference numbers (*t*_Li⁺_ < 0.5) [9–11]. This transport imbalance induces severe concentration polarization at Li metal anode interfaces due to the fast-moving anion accumulation, which cannot be compensated for by the slow Li^+^ supply, resulting in a space charge field, uneven ion flux, and dendrite Li growth [12,13]. These instabilities accelerate interfacial passivation and internal short circuits, often leading to earlier failure of polymer-based SSLMBs than their liquid counterparts.

To alleviate these challenges, immobilizing anions and maximizing the current fraction carried by Li^+^ have become compelling design strategies [14,15]. Single-ion-conducting polymer electrolytes (SICPEs) embody this concept by covalently tethering anions to the polymer backbone, thereby restricting their movement and ensuring that Li^+^ becomes the primary charge carrier. In principle, this design enables *t*_Li⁺_ values close to unity, eliminates concentration gradients, and promotes uniform Li deposition [16,17], which is crucial for high-rate operation and dendrite suppression in lithium batteries. Recent studies have shown that the *t*_Li⁺_ value exceeds 0.9, verifying the feasibility of this approach. However, the strong Coulombic interactions between Li^+^ and immobilized anions, coupled with the rigidity of the ionic skeleton, greatly limit the mobility of Li^+^, resulting in insufficient room-temperature conductivity. Many SICPE matrices possess narrow electrochemical windows and insufficient oxidative stability, limiting their compatibility with high-voltage cathode materials [18–20]. Although the SICPE conceptually addresses the root cause of interfacial failure in SSLMBs, its practical application is still hindered by low conductivity and limited stability.

To address these constraints, composite electrolytes incorporating ordered porous frameworks have attracted increasing attention [21]. Covalent organic frameworks (COFs) with crystalline nanochannels and tunable chemistry act as ion redistributors that immobilize anions, creating continuous Li⁺ pathways and homogenizing interfacial fluxes [22–26]. Crown ethers are known for their ability to select complex alkali metal cations, providing a well-defined coordination environment that facilitates Li^+^ transport. The integration of crown ethers further enriches the functionality of COFs, as their cavity size and donor arrangement enable selective Li^+^ coordination [27,28]. Rationally tuning the size and binding strength of crown ethers provides a promising route to balance confinement and mobility, thus advancing COF-based polymer composites toward practical SSLMBs.

**Figure 1.**
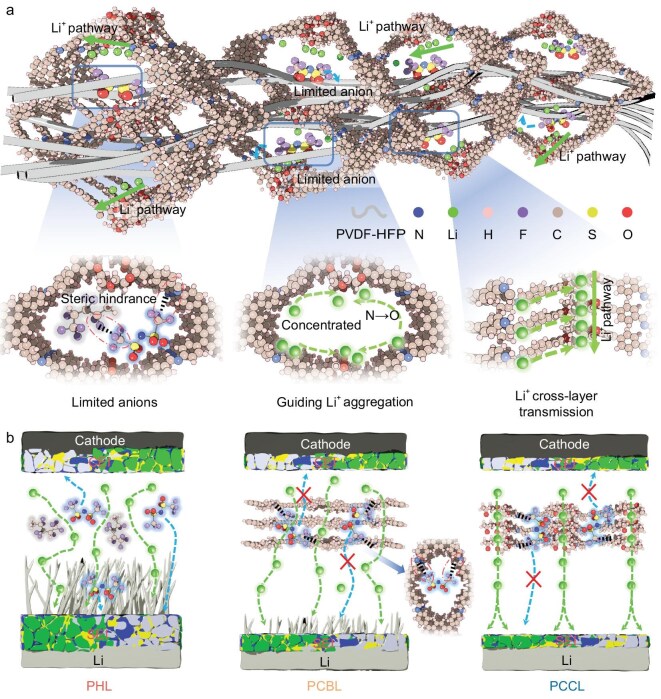
The regulatory mechanism of COF-Py-CE on (a) ionic migration in the PCCL solid-state electrolyte and on (b) interfacial chemistry.

Herein, we report a quasi-single-ion-conducting polymer electrolyte (QSICPE) based on a crown ether–functionalized COF. The imine groups in the COFs and fluorinated polymer domains immobilize anions (TFSI^−^) via electrostatic adsorption and steric confinement, while the crown ether oxygen dynamically coordinates Li^+^, enabling the anion to dissociate and undergo rapid interlayer hopping through ordered two-dimensional (2D) channels (Fig. 1). This dual regulation creates a Li^+^-preferential network with high conductivity (1.15 × 10^−3^ S cm^−1^), transference number of 0.91, and uniform interfacial flux, resulting in a thin and robust interphase. As a result, the symmetric cells can stably cycle for 1500 h at 0.5 mA cm^−2^, while the full cell retains 90% of its capacity after 2450 cycles, and the corresponding 1.2 Ah pouch cell maintains 95% capacity after 100 cycles. These results demonstrate that the crown ether–COF strategy can simultaneously immobilize anions and accelerate Li^+^ transport, providing a practical approach for high-performance SSLMBs.

## RESULTS AND DISCUSSION

### Synthesis and characterization of the functionalized COF conductor

**Figure 2.**
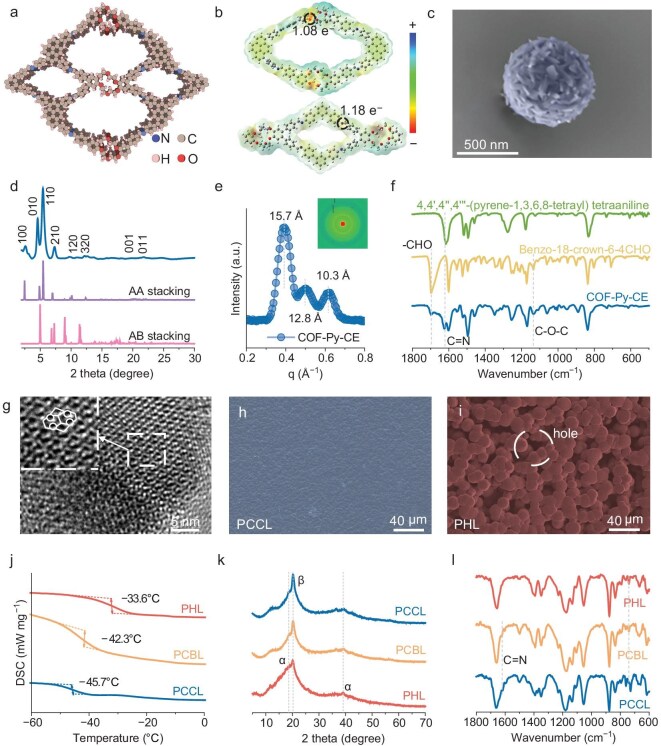
Synthesis of the PCCL electrolyte. (a) Top view of the space-filling model of COF-Py-CE. (b) ESP and Bader charge electron population. (c) SEM image of COF-Py-CE. (d) PXRD pattern of COF-Py-CE. (e) 2D SAXS pattern and pore size distribution of COF-Py-CE. (f) FTIR spectra of COF-Py-CE and its synthesis monomers. (g) HRTEM image of COF-Py-CE. Surface SEM images of (h) PCCL and (i) PHL. (j) DSC curves, (k) XRD patterns and (l) FTIR spectra of PCCL, PCBL and PHL.

A crystalline COF (COF-Py-CE) with abundant imine linkages, crown ether rings, and ordered porosity was synthesized by condensing 4,4′,4″,4^‴^-(pyrene-1,3,6,8-tetrayl)tetra aniline (Py) with a benzo-18-crown-6–4CHO (CE) monomer. For comparison, COF-Py-B was obtained by condensing Py with 4′,5′-bis(4-formylphenyl)- [1,1′:2′,1″-terphenyl]-4,4″-dicarbaldehyde, which features similar frameworks without crown ether sites for Li^+^ coordination (Fig. 2a and Figs S1, S2) [29]. Electrostatic potential (ESP) critically governs ion migration, with low-ESP regions facilitating Li^+^ transport and high-ESP regions favoring anion capture. Bader charge analysis revealed that the positively charged imine sites in both COFs attracted TFSI^−^ anions, promoting Li^+^ dissociation, whereas the crown ether units in COF-Py-CE provided negative ESP regions and oxygen bridges that accelerated Li^+^ transport across the polymer-COF interface (Fig. 2b and Figs S3, S4). High-resolution transmission electron microscopy (TEM) confirmed the presence of highly ordered 2D channels in both COFs (Fig. 2g and Fig. S5). COF-Py-CE exhibited three distinct pore sizes, whereas COF-Py-B exhibited two, and these channels provided accessible space to accommodate the polymer chains. Scanning electron microscopy (SEM) images showed that COF-Py-CE formed hollow spherical aggregates (Fig. 2c and Fig. S6), whereas COF-Py-B consisted of discrete dense solid spheres. Energy-dispersive X-ray spectroscopy (EDX) mapping confirmed the uniform distribution of all elements throughout the COF frameworks (Figs S7–S10), and powder X-ray diffraction (PXRD) confirmed the crystallinity with an AA-eclipsed stacking mode (Fig. 2d and Fig. S11), indicating coherent and well-ordered channel frameworks. COF-Py-CE shows three prominent diffraction peaks at 2.86°, 4.71° and 5.56°, corresponding to the (100), (010) and (110) planes, respectively. Small-angle X-ray scattering (SAXS) indicated that COF-Py-CE has three distinct pore sizes (1.57, 1.28 and 1.03 nm) and COF-Py-B has two predominant pore sizes (1.61 and 1.18 nm, Fig. 2e and Fig. S12). N_2_ adsorption analysis (BET) showed that COF-Py-CE exhibits a larger specific surface area (1344 m^2^ g^−1^) than COF-Py-B (795 m^2^ g^−1^, Fig. S13), further validating the pore size distributions with highly ordered porous architectures. Fourier transform infrared (FT-IR) spectroscopy showed a new peak at 1623 cm^−1^ (C=N stretching) with the −CHO aldehyde peak at 1695 cm^−1^ disappearing (Fig. 2f and Fig. S14), confirming that imine linkages have formed. Solid-state ^13^C NMR spectroscopy confirmed the formation of the COF (Fig. S15), showing an imine carbon resonance at 159 ppm, crown ether carbon ~70 ppm, and additional peaks from aromatic carbons. Thermogravimetric analysis (TGA) indicated high thermal stability >400°C (Fig. S16).

Composite solid electrolytes were then prepared by blending each COF with PVDF-HFP and LiTFSI, yielding PVDF-HFP@COF-Py-CE@LiTFSI (PCCL) and PVDF-HFP@COF-Py-B@LiTFSI (PCBL), alongside a COF-free polymer electrolyte (PVDF-HFP@LiTFSI, PHL). SEM images revealed that PCCL and PCBL membranes were thin (21.6 and 25.6 μm), dense, and uniform, whereas PHL was much thicker (63.1 μm) with an uneven and porous structure (Fig. 2h and i; Figs S17, S18). Corresponding EDX mapping images confirmed homogeneous element distribution in PCCL and PCBL, whereas the elemental signals of PHL were mostly confined to the polymer matrix (Figs S19–S21). Differential scanning calorimetry (DSC) analysis showed that the addition of COF reduced the glass transition temperature of PVDF-HFP (from −33.6°C in PHL to −45.7°C in PCCL and −42.3°C in PCBL, Fig. 2j), indicating that mobility of the polymer chains was enhanced and the barrier to Li^+^ migration was reduced. XRD analysis showed that COF induced the transition of PVDF-HFP from the α to β phase, which was attributed to the interaction between the ionic dipoles and the inhomogeneous COF surface ESP (Fig. 2k). These ion-dipole interactions create continuous Li^+^ conduction pathways along the polymer-COF interface, thereby enhancing the overall ionic conductivity of the membrane. FT-IR further confirmed weaker polymer–solvent interactions in PCCL, implying fewer [Li(DMF)_x_]^+^ complexes and more Li^+^ available for crown ether coordination. A greater fraction of Li^+^ remains free from solvent coordination and is hosted within the crown ether rings of COF-Py-CE, where the local electric fields and uniform ion flux promote homogeneous Li^+^ deposition on the electrode. This was further confirmed by TGA (Fig. S22), which showed that the solvent retention in PCCL (1.2% at 100°C) was lower than that of PCBL (2.3%) and PHL (3.0%). The crown ether–mediated Li⁺ hosting combined with reduced solvent coordination resulted in more efficient ion transport and more uniform Li deposition in PCCL.

### Migration mechanism of anions and cations

**Figure 3.**
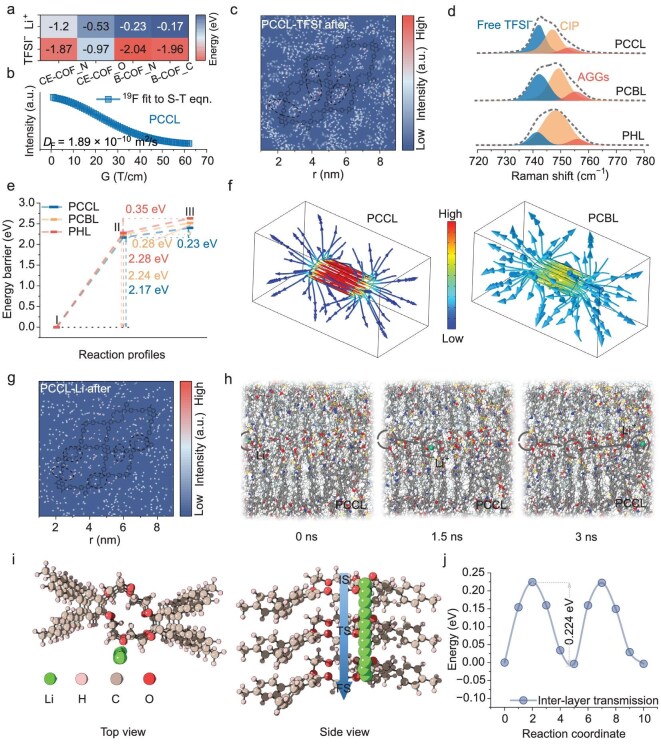
Simulation and analysis of the ion migration mechanism in PCCL. (a) The adsorption energy of Li^+^ and TFSI^−^ at different sites of COF-Py-CE and COF-Py-B within the electrolyte. (b) ^19^F NMR intensity and fitted decay curves used to calculate the diffusion coefficient of TFSI^−^ anions in PCCL. (c) Density distribution map of TFSI^−^ in PCCL after simulation. (d) Raman spectra of PCCL, PCBL and PHL. (e) Li^+^ migration energy barriers in PCCL, PCBL and PHL. (f) FEM simulations of Li^+^ migration within the COF rings in PCCL and PCBL. (g) Density distribution map of Li^+^ in PCCL after simulation. (h) Simulation snapshots of Li^+^ interlayer migration in COF-Py-CE within the PCCL electrolyte. (i) Schematic model of Li^+^ interlayer migration in COF-Py-CE. (j) Energy values at each point during Li^+^ interlayer migration.

Electrostatic potential mapping shows positively charged regions at the imine (C=N) sites and negatively charged regions at the crown ether oxygen sites. Density functional theory (DFT) calculations reveal that the C=N sites in PCCL strongly adsorb TFSI^−^ (−1.87 eV) and promote Li^+^ dissociation, while crown ether sites provide moderate binding that anchors Li^+^ (−0.53 eV) without blocking their subsequent migration (Fig. 3a and Fig. S23). In contrast, the C=N sites bind TFSI^−^ even more strongly (−2.04 eV) in PCBL. Li^+^ lacks additional binding sites once dissociated in PCBL without crown ether rings and thus primarily diffuses through the polymer matrix [30]. To validate the DFT prediction of anion immobilization, TFSI^−^ self-diffusion was measured by diffusion-ordered NMR spectroscopy (DOSY, Fig. 3b and Fig. S24). The ^19^F DOSY signal (78 ppm) shows that the diffusion coefficients of TFSI^−^ in PCCL, PCBL and PHL are 1.89 × 10^−10^ m^2^ s^−1^, 2.32 × 10^−10^ m^2^ s^−1^, and 4.45 × 10^−10^ m^2^ s^−1^, respectively, which indicate that the migration rate of TFSI^−^ in COF-containing electrolytes is significantly reduced compared to the COF-free electrolytes, confirming the effective immobilization of the anion [31]. From the molecular dynamics (MD) simulations (Fig. 3c and Figs S25, S26), TFSI^−^ anion aggregates around the imine groups in COF-Py-CE, and PVDF-HFP polymer chains had infiltrated into COF pores, which enhances fluorophilic interactions between TFSI^−^ and the C-F segments in polymer. Combined with steric confinement from COF channels and strong anion adsorption at C=N sites, these effects impose an effective spatial restriction on anion diffusion. PCBL shows a similar anion aggregation effect (Fig. S27), but not as pronounced as that in PCCL. In sharp contrast, TFSI^−^ in PHL remains highly mobile and uniformly dispersed throughout the electrolytes (Fig. S28) owing to the absence of COF-induced confinement of trapped anions [32]. Raman spectra further confirmed the anion-trapping effect in PCCL (Fig. 3d), revealing the highest fraction of free (uncoordinated) TFSI^−^ anions and the lowest fractions of contact ion pairs (CIP: Li^+^·TFSI^−^) and aggregates (AGG: Li^+^(TFSI^−^)_n_). This suggests that most TFSI^−^ anions are immobilized near imine sites and C-F groups through electrostatic attraction and steric confinement, thereby releasing more Li^+^ ions as active charge carriers. In contrast, PCBL and PHL show a gradual decrease in free anions and an increase in CIP/AGG, which is consistent with the weakening of the inhibitory effect on anion mobility [33]. These results highlight the critical role of COF-Py-CE fillers in immobilizing TFSI^−^, regulating the local ionic environment, and establishing a more favorable Li^+^ transport network at the polymer–COF interface.

Since suppressing TFSI^−^ releases more free Li^+^, it is crucial to explore the migration process of Li^+^. The energy barrier of DFT calculations revealed a two-step pathway in PCCL: Li^+^ first detaches from PVDF-HFP chains (step I→II with a barrier of 2.17 eV) and then enters the COF-Py-CE framework (step II→III with a barrier of 0.23 eV, Fig. 3e). The total Li^+^ migration barrier in PCCL is ~2.40 eV, which is significantly lower than in PCBL (2.52 eV) and PHL (2.63 eV, Fig. S29) [34]. These results demonstrate that the introduction of COF reduces the energy cost of Li^+^ transport and provides multiple low-barrier pathways, while the crown ether unit further lowers the energy barrier and enhances ionic conductivity. Finite element modeling (FEM) shows that Li^+^ flux in PCCL is largely confined within COF-Py-CE and directed inward to the interior of the particles (Fig. 3f). Li^+^ leaks largely into the polymer for PCBL, and the diffusion in PHL is random and isotropic owing to the lack of structural guidance (Figs S30–S32) [35]. MD-derived Li^+^ density maps provide further insights into ion transport pathways (Fig. 3g and Fig. S33). In PCCL, Li^+^ accumulates near C=N sites and crown ether oxygen sites, showing a clear tendency for Li^+^ to migrate from C=N sites to adjacent crown ether sites. A small amount of residual Li^+^-DMF complexes ([Li(DMF)_x_]^+^) was rapidly liberated upon encountering crown ether oxygens, facilitating ion transport (Fig. S34). For PCBL, Li^+^ remains largely around imine sites within COF pores, without equivalent secondary hopping sites (Fig. S35), while in PHL, Li^+^ is randomly distributed and heavily coordinated by DMF, severely limiting its mobility (Fig. S36). Radial distribution function (RDF) and coordination number (CN) analyses further confirm that Li^+^ exhibits lower coordination with TFSI^−^ and DMF in PCCL and PCBL than in PHL (Figs S37–S39), where PCCL shows the lowest Li^+^-TFSI^−^/DMF coordination and predominant interactions with crown ether O and imine N sites, demonstrating a Li^+^ migration pathway transporting ions from C=N to the crown ether site. MD trajectory analyses reveal two Li^+^ migration modes in PCCL including interlayer hopping between COF layers through crown ether sites and in-plane diffusion along a single COF layer (Fig. 3h and Figs S40–S42). Over a 3 ns simulation for PCCL, interlayer hopping covers longer distances than in-plane diffusion, highlighting the efficiency of crown ether–mediated interlayer transport. In PCBL, both modes exist without a dominant pathway, resulting in inefficient Li^+^ transport. Transition-state calculations further elucidate the Li^+^ migration pathways and quantify their associated energy barriers [36]. In PCCL, Li^+^ first accumulates at C=N sites and then migrates toward neighboring crown ether sites with an energy barrier of 1.12 eV (Fig. S43), which is lower than hopping across the pore from one C=N site to another (1.35 eV, Fig. S44). The barrier for interlayer hopping mediated by crown ether oxygens is only 0.224 eV (Fig. 3i and j), which is much lower than the interlayer barrier at C=N sites (1.97 eV) and the intralayer (in-plane) migration across the crown ether ring (0.291 eV, Figs S45, S46), further confirming that the crown ether–facilitated interlayer pathway is the faster Li^+^ transport route in PCCL. In contrast, in PCBL, the interlayer barrier (0.356 eV) is slightly higher than the in-plane barrier (0.335 eV) and far lower than the barriers associated with the imine-N sites (6.71 eV for cross-layer and 1.52 eV for in-plane migration, Figs S47–S49), indicating no preferred Li^+^ pathway and greater reliance on diffusion through the polymer matrix. These results confirm that exchange coordination between C=N and crown ether sites coupled with crown site–guided interlayer hopping constitutes the kinetically preferred Li^+^ migration pathway in PCCL. Macroscopically, the MD-derived Li^+^ self-diffusion coefficients confirm this mechanism (Fig. S50), where the Li^+^ self-diffusion coefficient in PCCL is 5.86 × 10^−10^ m^2^ s^−1^, much higher than PCBL (4.26 × 10^−10^ m^2^ s^−1^) and PHL (7.14 × 10^−11^ m^2^ s^−1^). Solid-state ^7^Li NMR spectroscopy shows a 0.24 ppm downfield shift in the Li^+^ resonance for PCCL relative to PCBL and PHL (Fig. S51), which is consistent with a significant enhancement in Li^+^ mobility [37]. These results demonstrate that the introduction of COF-Py-CE significantly improves ion transport in the polymer electrolyte, where the C=N groups electrostatically capture TFSI^−^ anions and release Li^+^ from the Li^+^-TFSI^−^ complex. At the same time, the crown ether oxygen atoms act as low-energy ‘stepping stones’ for Li^+^ hopping between COF layers, enabling a continuous and efficient Li^+^ conduction pathway at the polymer-COF interface.

### Enhanced ion transport and interfacial stabilization

**Figure 4.**
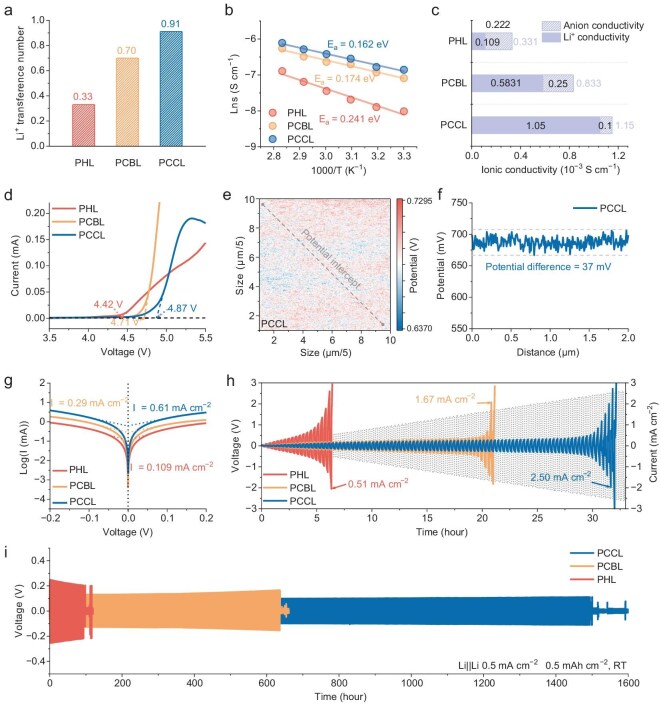
Electrochemical characterization of Li^+^ transport in PCCL. (a) Li^+^ transference numbers of PCCL, PCBL and PHL. (b) Arrhenius plots of PCCL, PCBL and PHL. (c) The actual Li^+^ conductivities of PCCL, PCBL and PHL. (d) LSV curves. (e) Potential distribution on the surface of PCCL in KPFM mode. (f) The potential difference on the surface of PCCL. (g) Tafel plots for Li||Li symmetric cells with PCCL, PCBL and PHL electrolytes. (h) Critical current density (CCD) test. (i) Long-term cycling performance of Li||Li symmetric cells using PCCL, PCBL and PHL at 0.5 mA cm^−2^ and 0.5 mAh cm^−2^.

As shown in Fig. 4a and Fig. S52, PCCL delivers a *t*_Li⁺_ of 0.91, which is much higher than that of PCBL (0.70) and PHL (0.33), reflecting the effective fixation of TFSI^−^ at the imine sites of COF and the spatial confinement in the fluorinated polymer domain. Electrochemical impedance spectroscopy further revealed that PCCL possesses high ionic conductivity of 1.15 × 10^−3^ S cm^−1^ at 30°C, which exceeds that of PCBL (8.33 × 10^−4^ S cm^−1^) and is four times that of PHL (3.31 × 10^−4^ S cm^−1^, Fig. 4b and Fig. S53). Notably, PCCL also shows a lower activation energy of 0.162 eV compared to PCBL (0.174 eV) and PHL (0.241 eV), which is consistent with the promotion of interlayer Li^+^ hopping via crown ether sites. Combining the conductivity and *t*_Li⁺_ (Fig. 4c), we obtain an effective Li^+^ conductivity of 1.05 × 10^−3^ S cm^−1^, which is almost twice as high as that of PCBL (5.83 × 10^−4^ S cm^−1^) and an order of magnitude higher than that of PHL (1.09 × 10^−4^ S cm^−1^) [38]. This confirms that the COF-Py-CE in PCCL simultaneously immobilizes TFSI^−^ and accelerates Li^+^ transport, yielding a markedly higher fraction of the current carried by Li^+^. Electrochemical stability was further investigated by linear sweep voltammetry (LSV, Fig. 4d), where the stability window is extended from 4.42 V for PHL to 4.87 V for PCCL, attributable to the efficient inhibition of interfacial side reactions via TFSI^−^ confinement. Kelvin probe force microscopy (KPFM) mapping revealed that the PCCL membrane has an extremely uniform surface potential with a total variation of only 37 mV across its surface (Fig. 4e, f and Fig. S54), while the PCBL and PHL membranes exhibit larger potential variations of 42 mV and 96 mV, respectively. The highly uniform surface potential of PCCL is due to the significantly reduced participation of anions in the system, which greatly mitigates parasitic reactions under high voltage, minimizes interfacial polarization, and suppresses local current hotspots, thereby helping to improve its oxidation stability [39]. Tafel plots further confirmed improved Li^+^ kinetics at the Li/electrolyte interface (Fig. 4g), with PCCL delivering a much higher exchange current density of 0.61 mA cm^−2^ compared with PCBL (0.29 mA cm^−2^) and PHL (0.109 mA cm^−2^) [40]. Meanwhile, the Li/Cu half-cell also exhibited enhanced Coulombic efficiency (Fig. S55).

This enhanced Li^+^ transport and interfacial stability translate into significantly improved critical current density (CCD) and cycling performance. Li/PCCL/Li symmetric cells sustain a CCD value of 2.5 mA cm^−2^, which is much higher than those with PCBL (1.67 mA cm^−2^) and PHL (0.51 mA cm^−2^, Fig. 4h). At 0.5 mA cm^−2^ and 0.5 mAh cm^−2^, PCCL-based symmetric cells can be stably cycled for >1500 h with minimal polarization (Fig. 4i), while PHL-based cells short within ~100 h with high polarization due to dendritic failure. Incorporating the COF framework not only regulates ion transport but also improves the mechanical properties of the membranes. As shown in Fig. S56, PCCL and PCBL exhibit higher tensile strength while maintaining sufficient ductility. The atomic force microscope (AFM) Young’s modulus distribution maps (Fig. S57) further reveal a more homogeneous mechanical landscape, indicating good COF dispersion within the electrolyte and a more continuous polymer/COF interphase. These features are beneficial for maintaining intimate electrode contact and alleviating stress accumulation during cycling, consistent with the enhanced CCD and the long-term cycling stability of Li||Li symmetric cells. These results highlight that the well-ordered COF channels homogenize Li^+^ flux during continuous plating/stripping processes, while TFSI^−^ immobilization suppresses side reactions and promotes the formation of a thinner and denser solid electrolyte interphase (SEI) layer [41]. Meanwhile, stable long-term cycling helps to form a passivated and self-limiting interface, thereby stabilizing the interface while maintaining an intact ion transport network in the bulk electrolyte. These factors work together to achieve a smoother Li deposition behavior, a more stable Li/electrolyte interface, and improved cycling life.

### Structural stability and interfacial evolution

**Figure 5.**
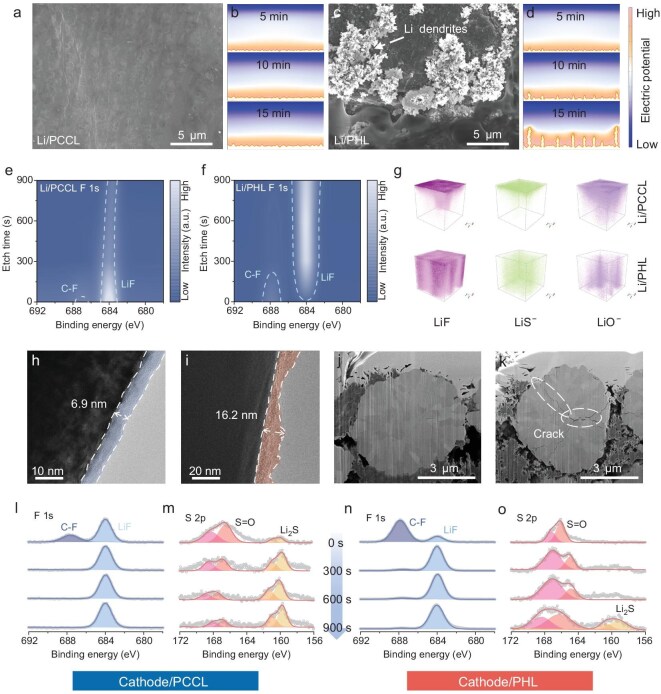
(a and c) SEM images of the Li metal anode surface with PCCL (a) and PHL (c) electrolytes. (b and d) FEM simulations of dendrite growth and current density variations on the Li metal anode surface with (b) PCCL and (d) PHL. (e and f) 2D XPS analysis of the F 1s region on the surface and depth profile of Li/PCCL (e) and Li/PHL (f) as a function of etching time. (g) Representative SEI components on Li/PCCL and Li/PHL surfaces reconstructed by 3D TOF-SIMS. (h and i) TEM images of the CEI layer on the NCM523 cathode surface using PCCL (h) and PHL (i) electrolytes. (j and k) Focused ion beam (FIB)-etched cross-sectional cracking in the NCM523 cathodes after cycling with PCCL (j) and PHL (k) electrolytes. (l–o) XPS depth-etched spectra of F 1s and S 2p on the surface of the NCM523 cathode after cycling with PCCL (l and m) and PHL (n and o) electrolytes.

To reveal the origin of the enhanced electrochemical performance, we investigated the structural evolution of the SEI and cathode-electrolyte interphase (CEI). SEM images reveal a sharp contrast in the morphology of the cycled Li metal anode, where PCCL exhibits a smooth and dense surface without any dendrite structure. In contrast, PHL shows extensive dendritic growth and ‘dead’ Li deposits, and PCBL displays moderate dendrite formation (Fig. 5a, c and Fig. S58). The Li^+^ deposition process was further simulated using FEM by monitoring the current density and potential on the Li surface. For PCCL, the current density remains uniformly distributed on the Li surface without hot spots or dendrite initiation during the electroplating process. This uniformity arises from the crown ether network, which homogenizes Li^+^ flux, shortens diffusion distances, and ensures dendrite-free deposition. In contrast, PCBL and PHL exhibit highly inhomogeneous current distributions within minutes, generating local hotspots and large potential gradients that drive rapid dendrite nucleation (Fig. 5b, d and Fig. S59) [42–44]. These simulations are well consistent with the experimentally observed stable Li surface in PCCL and severe dendritic growth in PHL. The chemical composition of the SEI was further investigated by X-ray photoelectron spectroscopy (XPS). In the F 1s spectra (Fig. 5e, f and Fig. S60), a peak at 687 eV corresponding to C-F bonds from PVDF-HFP or LiTFSI indicates residual organic fluorine in the SEI. For the cycled Li metal anode with PCCL, this C-F signal is much weaker than that of PCBL or PHL, which is consistent with the inhibition of TFSI^−^ degradation by PCCL. The peak located at 684 eV corresponds to LiF. For PCCL, the LiF signal is relatively strong at the surface and decreases gradually with depth, suggesting that any decomposition of TFSI^−^ or polymer is confined to a thin outer layer. The higher LiF intensity also suggests that the thin SEI formed for PCCL is enriched in inorganic species. The depth profiling for PCCL confirms that the corresponding SEI layer has the highest LiF and lowest C-F signals at the surface (Fig. S61), which decreases during sputtering, suggesting that the beneficial inorganic compounds are enriched in the outermost SEI layer [45]. In the S 2p spectrum, the peak near 168 eV corresponds to organic sulfur species from TFSI^−^ and one near 160 eV corresponds to the presence of Li_2_S. In the O 1s spectrum, peaks appearing at ~530 eV, 529.5 eV and 527 eV are attributed to organic oxygen species (ROLi or residual TFSI^−^), Li-OR and Li_2_O species. Compared with PCBL and PHL (Figs S62–S64), only PCCL detects the Li_2_O signal at 527 eV. This exclusive Li_2_O formation likely originates from interfacial reactions of crown ether oxygen atoms during long-term cycling, which consume organic O sites while enriching the SEI with inorganic species [46]. However, the consumption of these oxygen atoms arises from local interfacial transformations of the crown ether moiety, rather than from the overall breakdown of the COF transport network. The coexistence of Li_2_O with LiF and Li_2_S indicates that PCCL is beneficial for the formation of a thinner, denser, and inorganic-rich SEI layer on the surface of Li metal anodes.

Time-of-flight secondary ion mass spectrometry (TOF-SIMS) was further used to reconstruct the 3D distribution of SEI species, including LiF (LiF_2_^−^), Li_2_O (LiO^−^) and Li_2_S (LiS^−^). As shown in Fig. 5g and Fig. S65, compared with the PCBL and PHL, the cycled Li metal anode with PCCL contains markedly higher levels of LiF, Li_2_S, and especially Li_2_O, and the intensity of these inorganic species remains almost constant with the sputtering depth, indicating a uniform SEI composition throughout the thickness (Fig. S66). This uniformity contrasts with the gradient distribution typically observed in thicker SEIs and emphasizes that PCCL produces an improved inorganic-rich interphase, which is consistent with the XPS results. Beyond the anode side, cathode degradation was also probed. The conversion of layered LiNi_0.5_Co_0.2_Mn_0.3_O_2_ (NCM523, R3m) to rock-salt NiO (Fm3m) is the primary cause of structural failure, as the NiO phase is electrochemically inert and ionically insulating [47]. TOF-SIMS analysis of the cathode surface revealed that the intensity of NiO_2_^−^ fragments (a hallmark of the NiO phase) was lowest in PCCL compared to PCBL and PHL (Figs S67, S68), suggesting that the PCCL-derived CEI layer effectively suppresses the formation of rock salt, thereby maintaining the integrity of the cathode structure and mitigating capacity fade.

As shown from the TEM images in Fig. 5h, i and Fig. S69, the NCM cathode with PHL exhibits a thick and irregular CEI layer of ~16 nm. In sharp contrast, the CEI formed in PCCL was much thinner (~6.9 nm) and exhibited a smooth and dense morphology, further confirming that PCCL effectively suppresses parasitic side reactions at the cathode surface. Cross-sectional focused ion beam (FIB) microscopy showed that NCM particles cycled with PCCL retained an intact internal structure without visible microcracks, whereas cathodes cycled with PCBL and PHL exhibited significant intergranular cracks (Fig. 5j, k and Fig. S70), highlighting the protective role of the PCCL-derived CEI layer in mitigating cathode structural breakdown. CEI composition was further probed by depth-profiling XPS. The PCCL-derived CEI exhibits stronger LiF signals than those from PCBL and PHL (Fig. 5l, n and Fig. S71a). Given the high interfacial energy and excellent electronic insulation of LiF, its enrichment is crucial for suppressing electron tunneling and improving stability. Also, the PCCL-derived CEI layer exhibits a higher Li_2_S content (Fig. 5m, o and Fig. S71b). TOF-SIMS depth mapping further confirms that LiF and Li_2_S are more uniformly distributed in PCCL, and their intensities gradually decrease with etching depth, indicating confinement within the outermost CEI layer (Figs S72, S73). NiF_3_-based reconstruction reveals that the contents of reduced Ni species (Ni^2+^ and Ni^0^) are much lower in PCCL compared with PCBL and PHL (Figs S74, S75), further demonstrating the suppression of NiO formation in rock salt [48–50]. These results indicate that PCCL reconstructs the cathode-side interfacial microenvironment by confining TFSI^−^ near the C=N sites and fluorinated PVDF-HFP domains, while maintaining preferential Li^+^ transport through dynamic crown ether/imine coordination. This suppresses anion-driven polarization and local potential hot spots, thereby mitigating continuous oxidative decomposition of organic components and guiding the early self-limiting formation of a thin and inorganic-rich CEI layer at the interface. Therefore, the dense inorganic CEI layer passivates the cathode surface, protects NCM from phase-transition–induced degradation, and inhibits further electrolyte decomposition during long-term cycling.

### Electrochemical performance

**Figure 6.**
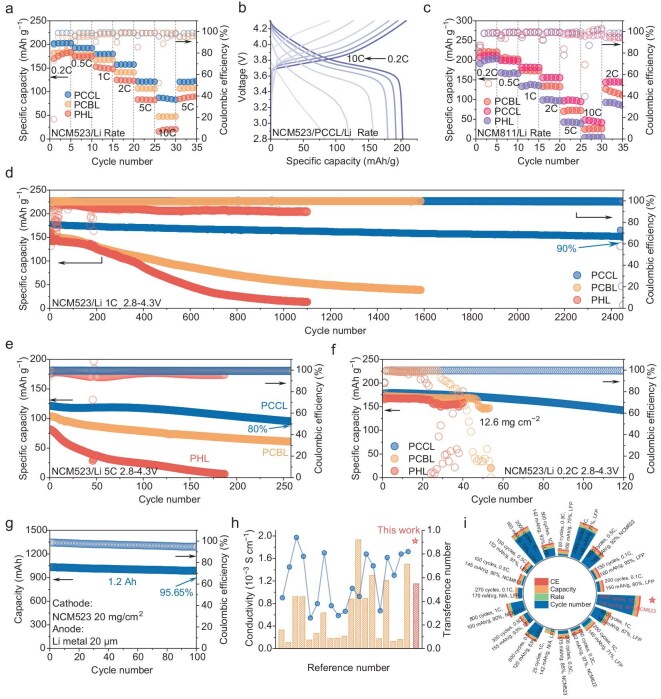
(a) Cycling performance of NCM523//Li cells at different rates. (b) Charging–discharging curves of NCM523/PCCL/Li cell under different rates. (c) Cycling performance of NCM811//Li cells at different rates. (d) Long-term cycling stability of NCM523/PCCL/Li, NCM523/PCBL/Li and NCM523/PHL/Li cells at 1.0 C rate at room temperature. (e) Long-term cycling stability of NCM523/PCCL/Li, NCM523/PCBL/Li and NCM523/PHL/Li cells at 5.0 C rate at room temperature. (f) Cycling stability of the NCM523//Li cells with an active material loading of 12.6 mg cm^−2^. (g) Cycling stability of the NCM523/PCCL/Li solid-state pouch cell at 0.1 C. (h and i) Comparative studies on the application of COF materials in polymer solid electrolyte systems, focusing on ionic conductivity and Li^+^ transference number (h), as well as cycling performance (i).

To assess the practical applicability of the proposed QSICPE, SSLMBs with NCM523 and LiNi_0.8_Co_0.1_Mn_0.1_O_2_ (NCM811) were assembled and tested at room temperature. As shown in Fig. 6a, the NCM523/PCCL/Li cell delivered reversible specific capacities of 202.7, 191.9, 179.8, 157.8, 120.8, and 82.6 mAh g^−1^ at rates from 0.2 C to 10.0 C (1.0 C = 200 mAh g^−1^), with full capacity recovery when the rate returned to 0.2 C. In contrast, PCBL- and PHL-based cells exhibit a low capacity of only 47.2 and 20.5 mAh g^−1^ at 10.0 C. Corresponding charging-discharging voltage profiles of the NCM523/PCCL/Li cell also maintain a stable voltage plateau with minimal polarization, highlighting its excellent kinetic stability (Fig. 6b and Fig. S76). Similar behavior was observed with NCM811 (Fig. 6c and Fig. S77), where NCM811-based cells achieved 211.2, 199.6, 181.4, 155.6, 97.1 and 48.1 mAh g^−1^ from 0.2 C to 10.0 C, whereas PCBL- and PHL-based cells showed near-complete capacity fading at high rates.

Long-term cycling further highlighted the durability of PCCL. At a cutoff voltage of 4.3 V, the NCM523/PCCL/Li cell retains 151.2 mAh g^−1^ after 2450 cycles at 1.0 C, corresponding to 90% capacity retention with an ultralow per-cycle decay of 0.004% (Fig. 6d). In contrast, the capacity of the PCBL-based cell drops to 38.1 mAh g^−1^ after 1600 cycles while the PHL-based cell delivers a capacity of 12.3 mAh g^−1^ after 1100 cycles. The voltage-capacity curves confirmed that the polarization in PCCL was still negligible even after 2400 cycles (Fig. S78). Similarly, the NCM811/PCCL/Li cell maintains 88.0% capacity retention after 350 cycles with a specific capacity of 149.8 mAh g^−1^ at 2.0 C (Figs S79, S80), while the NCM523/PCCL/Li cell delivers a specific capacity of 95.2 mAh g^−1^ with a capacity retention of 80% after 250 cycles at 5.0 C without significant polarization (Fig. 6e and Fig. S81). For the high-loading electrode (12.6 mg cm^−2^), the NCM523/PCCL/Li cell delivers a specific capacity of 142.8 mAh g^−1^ after 120 cycles at 0.2 C (Fig. 6f and Fig. S82). Furthermore, a 1.2 Ah NCM523/PCCL/Li pouch cell was fabricated to investigate practical feasibility. As shown in Fig. 6g and Fig. S83, the pouch cell delivers a reversible capacity of 978 mAh with an energy output of 3849 mWh (corresponding to 312 Wh kg^−1^) after 100 cycles at 0.2 C, with an impressive capacity retention of 95.65%, demonstrating excellent scalability and stability. These results highlight that the ordered 2D Li^+^ transport channels and strong anion confinement in PCCL synergistically accelerate Li^+^ migration and stabilize the interface, enabling high rate capability, long cycle life, and practical scalability. Compared with previously reported COF-based polymer electrolytes, PCCL performs best in terms of effective *σ*_Li⁺_, *t*_Li⁺_, and long-term durability (Fig. 6h and i).

## CONCLUSIONS

In summary, we incorporate crown ether–functionalized COFs into a fluorinated polymer matrix to develop a quasi-single-ion conducting polymer electrolyte, achieving simultaneous enhancement of Li^+^ transport and interfacial stability. The C=N bonds and fluorinated polymer domains electrostatically adsorb and spatially confine anions, while C=N sites and crown ether oxygen separate Li^+^ from its counterions and mediate dynamic exchange coordination. This dual regulation enables rapid interlayer Li^+^ hopping along ordered covalent organic framework nanochannels with a room-temperature ionic conductivity of 1.15 × 10^−3^ S cm^−1^ and a Li^+^ transference number of 0.91. The resulting Li^+^-preferential transport network ensures dendrite-free cycling for 1500 h at 0.5 mA cm^−2^, and sustains a critical current density value of 2.5 mA cm^−2^. The full cells retain 90% capacity after 2450 cycles at 1.0 C and 1.2 Ah pouch cells maintain 95% capacity after 100 cycles, providing a practical path to achieve high-performance solid-state batteries.

## MATERIALS AND METHODS

### Synthesis of COF-Py-CE

4,4′,4″,4″′-(pyrene-1,3,6,8-tetrayl)tetraaniline (0.024 mmol, 13.6 mg), benzo-18-crown-6–4CHO (0.024 mmol, 18.64 mg), 1 mL of o-dichlorobenzene (o-DCB), 1 mL of 1,4-dioxane, and 0.2 mL of 6 M aqueous acetic acid were added into a 10 mL Pyrex tube. The tube was rapidly frozen at 77 K, degassed by three freeze-pump-thaw cycles, and then sealed. The reaction mixture was heated at 120°C for 3 d. The resulting powder was collected, washed sequentially with N, N-dimethylacetamide (DMAC), tetrahydrofuran (THF), and methanol, and then vacuum-dried at 100°C for 12 h to afford the corresponding yellow powder in ~88% isolated yield.

### Synthesis of COF-Py-B

4,4′,4″,4″′-(pyrene-1,3,6,8-tetrayl)tetraaniline (0.024 mmol, 13.6 mg), 4′,5′-bis(4-formylphenyl)- [1,1′:2′,1″-terphenyl]-4,4″-dicarbaldehyde (0.024 mmol, 11.87 mg), 1 mL of o-DCB, 1 mL of 1,4-dioxane, and 0.2 mL of 6 M aqueous acetic acid were added into a 10 mL Pyrex tube. The tube was rapidly frozen at 77 K, degassed by three freeze-pump-thaw cycles, and sealed under vacuum. The reaction mixture was then heated at 120°C for 3 d. The resulting powder was collected, washed sequentially with DMAC, THF, and methanol, and finally vacuum-dried at 100°C for 12 hours to afford the corresponding bright yellow powder in ~90% isolated yield.

### Synthesis of PVDF-HFP@COF-Py-CE@LiTFSI (PCCL) electrolyte membranes

For the polymer composite matrix, 0.5 g of PVDF-HFP particles (Mw = 450 000, Sigma) and 0.5 g of LiTFSI were mixed with 10 wt% of COF-Py-CE in DMF solvent. After stirring at 60°C, the resulting homogeneous slurry was uniformly cast onto a polytetrafluoroethylene (PTFE) plate using a 50 μm doctor blade, followed by drying in a vacuum oven at 60°C for 24 h. The final COF content in the composite membrane is ~9.6–9.7 wt%.

### Synthesis of PVDF-HFP@COF-Py-B@LiTFSI (PCBL) electrolyte membranes

For the polymer composite matrix, 0.5 g of PVDF-HFP particles (Mw = 450 000, Sigma) and 0.5 g of LiTFSI were mixed with 10 wt% of COF-Py-B in DMF solvent. After stirring at 60°C, the resulting homogeneous slurry was uniformly cast onto a PTFE plate using a 50 μm doctor blade, followed by drying in a vacuum oven at 60°C for 24 h.

### Synthesis of PVDF-HFP@LiTFSI (PHL) electrolyte membranes

For the polymer composite matrix, 0.5 g of PVDF-HFP particles (Mw = 450 000, Sigma) and 0.5 g of LiTFSI were mixed in DMF solvent. After stirring at 60°C, the resulting homogeneous slurry was uniformly cast onto a PTFE plate using a 50 μm doctor blade, followed by drying in a vacuum oven at 60°C for 24 h.

## Supplementary Material

nwag098_Supplemental_File
