# Supplementary material for: Crown-ether-programmed covalent organic framework nanochannels enable quasi-single-ion conductivity for solid-state lithium metal batteries
Source: Natl Sci Rev. 2026 Feb 13;13(7):nwag098. doi: 10.1093/nsr/nwag098 (PMC13105168; doi:10.1093/nsr/nwag098)
Supplement: nwag098_Supplemental_File [file nwag098_supplemental_file.pdf]

SUPPLEMENTARY DATA

Crown-Ether-Programmed Covalent Organic Framework Nanochannels Enable Quasi-Single-Ion Conductivity for Solid-State Lithium Metal Batteries

Yihang Nie[1], Shibin Li[2], Tingzhou Yang[4,*], Longjie He[2], Guo Feng[1], Yiting Shao[1], Qingying Li[1], Jiawei He[1], Mingliang Jin[1], Dan Luo[3,*], Xin Wang[2,5,*], Zhongwei Chen[3,*]

[1]Guangdong Provincial Key Laboratory of Optical Information Materials and Technology, South China Academy of Advanced Optoelectronics, International Academy of Optoelectronics at Zhaoqing, South China Normal University, Guangzhou 510006, China

[2]Institute of Carbon Neutrality, Zhejiang Wanli University, Ningbo 315100, China

[3]State Key Laboratory of Catalysis, Dalian Institute of Chemical Physics, Chinese Academy of Sciences, Dalian 116023, China

[4]School of Chemistry and Chemical Engineering, Nantong University, Nantong 226019, China

[5]Ningbo Key Laboratory of High Energy Density Battery, Yuyao Innovation Institute, Zhejiang Wanli University, Ningbo 315100, China

***Corresponding authors.** E-mails: tzyang@ntu.edu.cn; luodan@dicp.ac.cn; wangx@zwu.edu.cn; zwchen@dicp.ac.cn

Experimental Section

Materials.

Benzo-18-crown-6-4CHO, 4,4',4'',4'''-(pyrene-1,3,6,8-tetrayl) tetra aniline, 4',5'-bis(4-formylphenyl)-[1,1':2',1''-terphenyl]-4,4''-dicarbaldehyde, purchased from Jilin Chinese Academy of Sciences-Yanshen Technology Co., Ltd. 1,2-Dichlorobenzene (o-DCB), acetic acid, 1,4-dioxane, N,N-dimethylacetamide (DMAC), and N,N-dimethylformamide (DMF), tetrahydrofuran (THF), N-methyl-2-pyrrolidone (NMP) and methanol were all purchased from Aladdin Chemistry Co., Ltd. LiTFSI and lithium foils were purchased from DodoChem. Poly(vinylidene fluoride-co-hexafluoropropylene) (PVDF-HFP) was purchased from Sigma-Aldrich. Poly(vinylidene fluoride) (PVDF), Super P, NCM523, and NCM811 were purchased from Guangdong Canrd New Energy Technology Co., Ltd. All reagents were used as received without further purification.

Material Characterizations

X-ray diffraction (XRD) patterns of the powders and electrolyte membranes were measured using a Bruker D8 diffractometer (Cu Kα, λ = 1.5418 Å). The pore structure of COF materials was characterized by small-angle X-ray scattering (SAXS, Xenocs Xeuss 3.0). The pore size was calculated based on the function of the scattering vector q ($q = 4\pi sin\theta/\lambda$) and the reciprocal space relation ($d = 2\pi/q$). The specific surface area was calculated using the Brunauer Emmett Teller (BET) method, while the pore volume was derived from the adsorption isotherms based on the nonlocal density functional theory (NLDFT) model. X-ray photoelectron spectroscopy (XPS, Thermo Scientific ESCALAB Xi^+) was used to determine the chemical states of the compounds and the SEI components. The spectra were obtained using an Al Kα (λ = 0.83 nm, hν = 1,486.7 eV) X-ray source operating at 2 kV and 20 mA. Thermogravimetric analysis (TGA) was conducted with a Mettler TGA 2 instrument to calculate the carbon content of thermal samples in an air atmosphere. Raman spectra were recorded using a Jobin Yvon Labram HR-800 spectrometer. Mechanical testing was carried out using an Instron ElectroPuls E1000 machine at a strain rate of 2 mm per minute. The morphology of the electrolyte membranes was examined using scanning electron microscopy (SEM, JEOL 7001F) coupled with an energy-dispersive X-ray (EDX) spectrometer. Fourier-transform infrared (FT-IR) spectra were

recorded within the range of 500-4000 cm^{-1} using a Thermo Scientific Nicolet iS50 spectrometer. Solid-state nuclear magnetic resonance (NMR) experiments were conducted at room temperature with a JNM-ECZ 600 MHz NMR spectrometer. Surface SEI components were analyzed using TOF-SIMS 5-100. The TOF-SIMS studies were performed on Li metal after 20 cycles, using a Cs^+ ion beam (1 keV ion energy, with a measured sample current of approximately 40 nA) to sputter a 50 × 80 μm^2 area centered on the analysis region. Depth profiling was conducted using a pulsed 30 keV $Bi1^+$ ion beam (20 ns) set to high current mode. Prior to XPS and TOF-SIMS characterization, the cycled Li metal was cleaned with propylene carbonate and dried in an argon-filled glovebox. Atomic force microscopy (AFM, Bruker, Dimension Icon) characterized the surface morphology of materials, and the corresponding KPFM mode characterized the surface potential of materials.

Electrochemical characterizations

The working electrodes were fabricated by thoroughly blending 80 wt% active material (NCM523, NCM811), 10 wt% carbon black, and 10 wt% polyvinylidene fluoride (PVDF) onto aluminum foil. For coin cells, high-loading cells, and pouch cells, the active material loadings were 2.0, 12.6, and 20.0 mg cm^{-2}, respectively. The cells were assembled using 450 μm Li foil and encapsulated in 2032-type coin cells, devoid of liquid electrolyte. In pouch cells, 20 μm Li foil coated on copper foil was employed, also without any liquid electrolyte. Ionic conductivity was assessed using electrochemical impedance spectroscopy (EIS), conducted with sandwiched lithium-ion blocking stainless steel electrodes. Measurements were performed over a frequency range of 1 MHz to 0.01 Hz with an AC amplitude of 10 mV, within a temperature range of 30°C to 70°C. The ionic conductivity (σ) was determined using the equation below:

$$\sigma = \frac{L}{SR_b}$$

where L represents the thickness of the electrolyte membrane, R_b is the bulk resistance, and S is the area of the stainless steel electrode (2.01 cm^2). The activation energy (E_a) was determined using the Arrhenius equation:

$$\sigma = \mathrm{A}e^{\frac{-E_a}{KT}}$$

where A is the pre-exponential factor and E_a is the activation energy for ion migration. Linear sweep voltammetry (LSV) was conducted using a Li/solid electrolyte/stainless steel disk at a

scan rate of 0.2 mV/s from 3.0 to 6.0 V (vs. Li/Li^+) at room temperature. The ion transference number (t^+) was measured by applied a 10 mV potential to Li symmetric cells using chronoamperometry and AC impedance spectroscopy. The transference number was calculated using the following equation:

$$t^+ = \frac{I_{ss}(\Delta V - I_0 R_0)}{I_0(\Delta V - I_{ss} R_{ss})}$$

I_0 and I_{ss} represent the initial current and the final current, respectively; ΔV is the applied bias voltage (10 mV); and R_0 and R_{ss} correspond to the initial resistance and the final resistance. The critical current density (CCD) was tested using a constant current cycling method, where the current was increased from 0.06 mA to 4.22 mA, the upper and lower voltage limits were preset to +1 V and –1 V. The electrochemical performance of the solid-state batteries was evaluated at room temperature within voltage ranges of 2.8 to 4.3 V using a Neware multichannel battery testing system. Cyclic voltammetry (CV) tests were performed at room temperature. The Li-ion diffusion coefficient was calculated using the Randles-Sevcik equation:

$$I_p = 2.687 \times 10^5 n^3 A D^{0.5} C w^{0.5}$$

where w is the scan rate, C represents the initial concentration of Li^+ in the positive electrode active material, D is the apparent Li-ion diffusion coefficient, A represents the electrode's projected area, n is the number of electrons involved in the transfer, and I_p represents the peak current intensity. Using the Aurbach CE protocol on the Neware battery test system, the Coulombic efficiency of lithium in various electrolytes was measured using Li||Cu cells. The cells were cycled for 10 cycles at a current density of 0.2 mA cm^{-2}, and the lithium was fully stripped to 1 V at the end. The Coulombic efficiency (CE) was determined using the following equation:

$$CE = \frac{(nQ_c + Q_s)}{(nQ_c + Q_r)}$$

where n is the number of cycles before the voltage spike, and Q_r, Q_c, and Q_s represent the total charge, the cycled charge, and the final stripping charge, respectively.

Computational methods and models

Density functional theory (DFT) calculations for all periodic systems were performed using the Vienna Ab Initio Simulation Package (VASP) coupled with the projector augmented

wave (PAW) method. The exchange-correlation functional was described using the generalized gradient approximation (GGA) proposed by Perdew-Burke-Ernzerhof (PBE). Van der Waals (vdW) interactions were calculated using Grimme's DFT-D3 correction scheme.[1,2] The kinetic energy cutoff of the plane wave basis set was set to 500 eV.[3] For geometric relaxation, the Brillouin zone was sampled using a 1×1×1 Γ-centered k-point grid. All slab models were constructed with a vacuum region of approximately 20 Å to ensure negligible lateral interactions between the periodic images of the adsorbate.[4] The bottom layer of the structure is constrained to its bulk lattice position, while all other atomic positions are fully relaxed until the total energy converges to a threshold of 1×10^{-6} eV and the average residual force on each atom is less than 0.02 eV $Å^{-1}$.[5] The adsorption energy (E_{ads}) is calculated according to the following formula:

$$E_{\text{ads}} = E_{\text{total}} - E_{\text{sub}} - E_{\text{adso}}$$

where E_{total} is the total energy of the optimized adsorbate-substrate complex, E_{sub} and E_{adso} are the energies of the isolated substrate and adsorbate, respectively, within the same unit cell. All calculations were performed using the same VASP parameters to ensure consistency. The minimum energy pathway for Li-ion diffusion was investigated using the climbing-image nudged elastic band (CI-NEB) method within the VASP framework. The initial reaction path, defined by linearly interpolated images between the initial and final configurations, was relaxed using an improved tangent scheme with the default spring force. Subsequently, the climbing-image algorithm was activated for the highest-energy image to precisely converge to the transition state (saddle point) by maximizing its energy along the path tangent. The diffusion energy barrier is defined as the energy difference between this saddle point and the initial stable state.

Classical molecular dynamics (MD) simulations were performed to investigate the hybrid electrolyte systems. Three model systems were constructed: PCCL, PCBL, and PHL. The PHL system consisted of 1418 LiTFSI, 856 PVDF-HFP, and 620 DMF molecules, contained within a cubic box with a side length of 9.82 nm. The PCBL system comprised 1430 LiTFSI, 863 PVDF-HFP, 624 DMF molecules, and a single COF-Py-B substrate in a cubic box with a 9.89 nm side length. The PCCL system was composed of 1418 LiTFSI, 856 PVDF-HFP, 620 DMF

molecules, and a single COF-Py-CE substrate, with a cubic box side length of 9.92 nm. In each case, the polymer was represented by a single PVDF-HFP chain segment, and the framework was modeled as a single COF unit. Initial configurations were generated using PACKMOL,[6] where all species were randomly placed within a cubic simulation box. The COF substrates were treated as rigid bodies, and their atomic positions were held constant throughout the simulations. All interactions were described by the General Amber Force Field (GAFF).[7] The force field encompasses both bonded (bonds, angles, and dihedrals) and non-bonded terms. The non-bonded interactions include van der Waals forces, modeled with a 12-6 Lennard-Jones potential, and electrostatic forces, described by Coulomb's law.

$$E_{LJ}(r_{ij}) = 4\varepsilon_{ij}\left[\left(\frac{\sigma_{ij}}{r_{ij}}\right)^{12} - \left(\frac{\sigma_{ij}}{r_{ij}}\right)^{6}\right]$$

$$E_c(r_{ij}) = \frac{q_i q_j}{4\pi\varepsilon_o\varepsilon_r r_{ij}}$$

In the equation, q_i and q_j are the atomic charges, r_{ij} is the interatomic separation, σ is the Lennard–Jones diameter, and ε is the well-depth parameter. For unlike pairs, van der Waals parameters were obtained using a geometric combining rule,

$$\sigma_{ij} = \sqrt{\sigma_{ii}\sigma_{ij}}$$

$$\varepsilon_{ij} = \sqrt{\varepsilon_{ii}\varepsilon_{jj}}$$

Subsequently, the systems were equilibrated first in the NVT ensemble for 1 ns, followed by the NPT ensemble for 10 ns, using a time step of 1.0 fs. This procedure ensured that equilibrium was reached at 333.15 K and 1.0 atm, as confirmed by stable box dimensions. Finally, a 5 ns production run was conducted in the NPT ensemble with a 1.0 fs time step to collect trajectories for analysis. Temperature and pressure were maintained using the Nosé-Hoover thermostat and the Parrinello-Rahman barostat, respectively. Throughout the simulations, the classical Newton's equations of motion were integrated using the velocity-Verlet algorithm. All MD simulations were performed using the GROMACS 2020.6 package.

For the finite-element simulations, a three-dimensional model comprising a COF domain and a polymer-electrolyte domain was built in COMSOL Multiphysics. Using the Transport of Diluted Species physics in the diffusion-only limit (i.e., neglecting convection and migration),

we simulated Li^+ diffusion under different open-boundary configurations. Specifically, the PCCL and PCBL models employed two open boundaries, whereas the PHL model employed three. The initial Li^+ concentration and diffusion coefficient within the COF framework were set to 1000 mol m^{-3} and 1 × 10^{-10} m^2 s^{-1}, respectively; in the electrolyte, the initial Li^+ concentration and diffusion coefficient were set to 0 mol m^{-3} and 1 × 10^{-11} m^2 s^{-1}, respectively. Two-dimensional finite element models were used to characterize ion transport and dendrite formation near the current collectors (PCCL, PCBL, and PHL solid electrolytes). Finite element simulations were performed using the tertiary current distribution module. The Nernst-Planck equation was used to evaluate lithium ion and anion transport. The computational domain was defined as a 5 × 10 μm electrolyte region. The upper and lower boundaries corresponded to the electrolyte and lithium metal, respectively. A current density of 0.5 mA cm^{-2} was applied, and the Butler-Volmer equation was used to solve the interfacial reaction kinetics. The ion transference numbers for PCCL, PCBL, and PHL were set to 0.91, 0.70, and 0.33, respectively, and the ionic conductivities were set to 1.15 × 10^{-3} S cm^{-1}, 8.33 × 10^{-4} S cm^{-1}, and 1.09 × 10^{-4} S cm^{-1}.

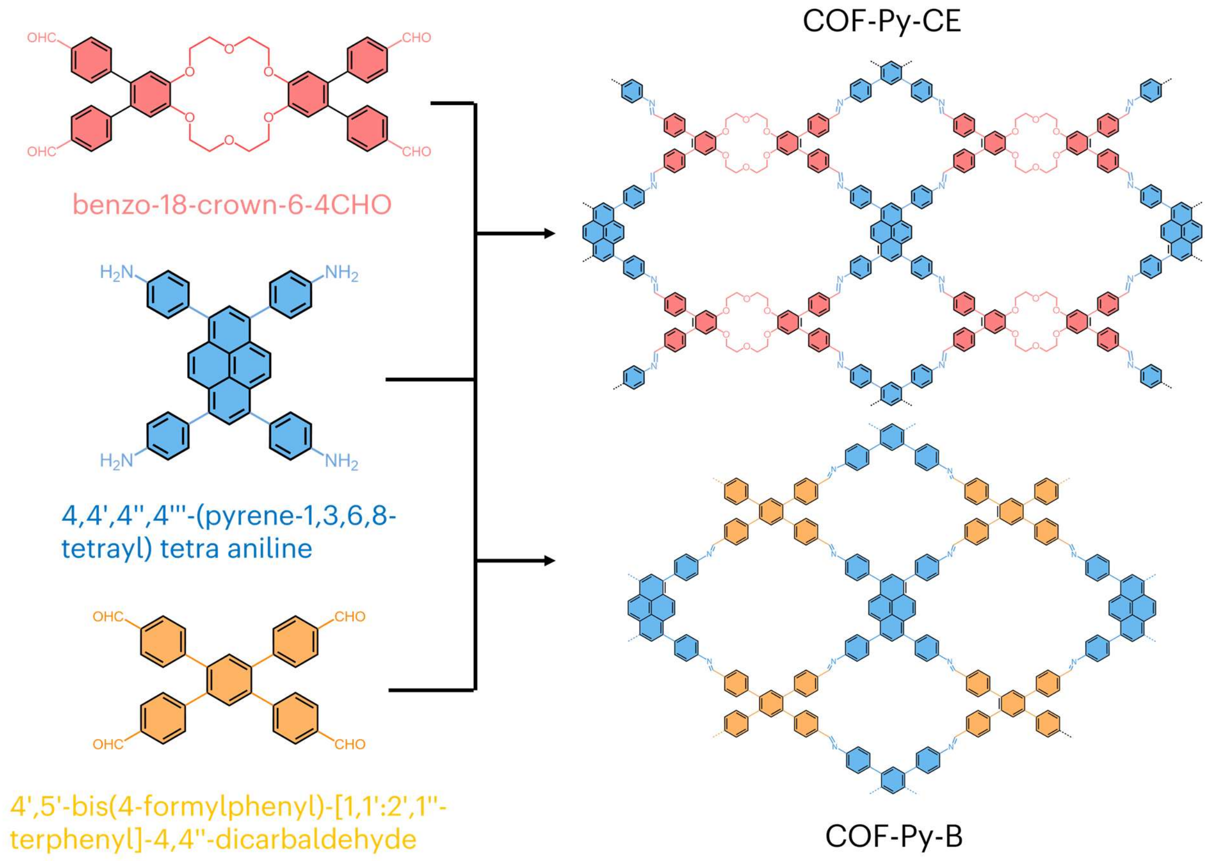

Figure S1. Synthetic routes and monomer presentation of COF-Py-CE and COF-Py-B.

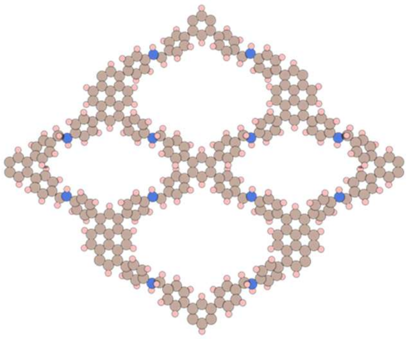

Figure S2. Top view of the space-filling model of COF-Py-B.

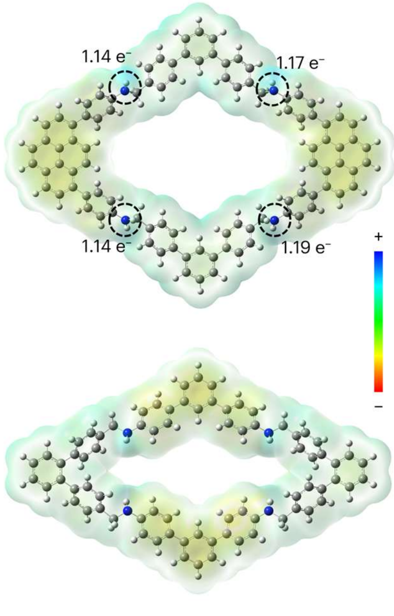

Figure S3. Electrostatic potential (ESP) and Bader charge electron population of COF-Py-B.

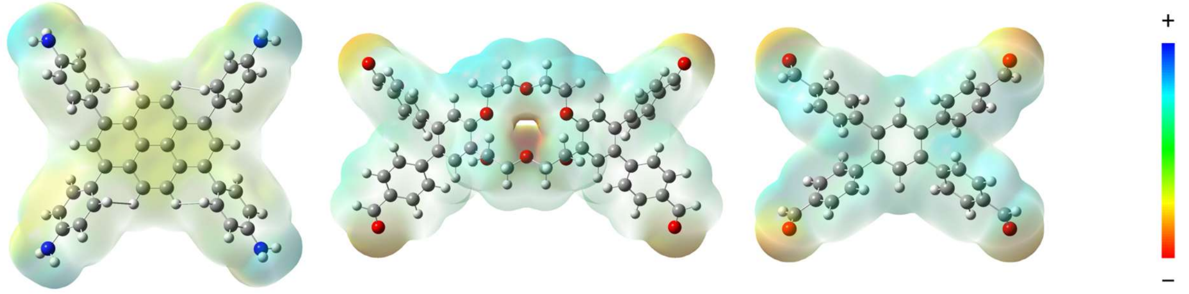

Figure S4. The electrostatic potential distribution maps of the three monomers.

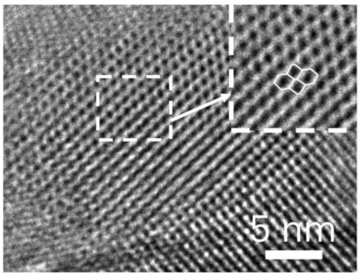

Figure S5. The TEM images of COF-Py-B and its corresponding pore size distribution.

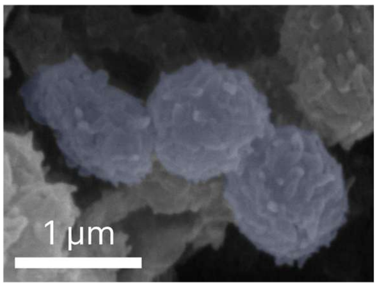

Figure S6. The SEM morphological characterization of COF-Py-CE.

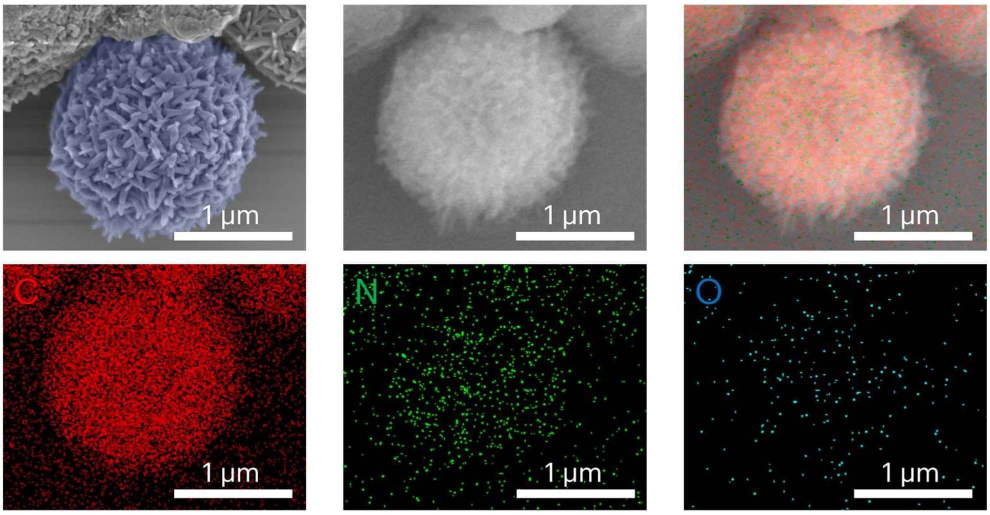

Figure S7. The EDS morphology and elemental distribution maps of COF-Py-CE.

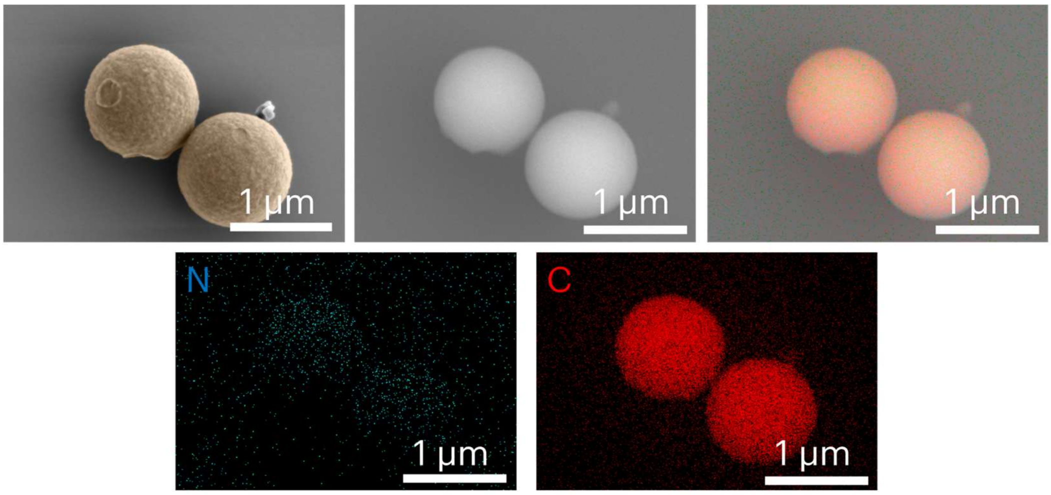

Figure S8. The SEM morphology and elemental distribution maps of COF-Py-B.

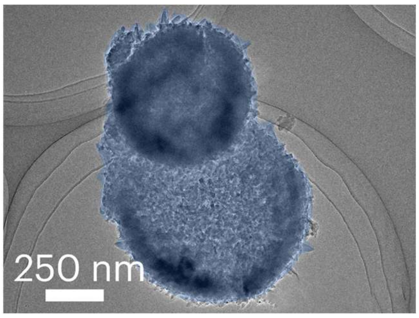

Figure S9. The TEM morphology COF-Py-CE.

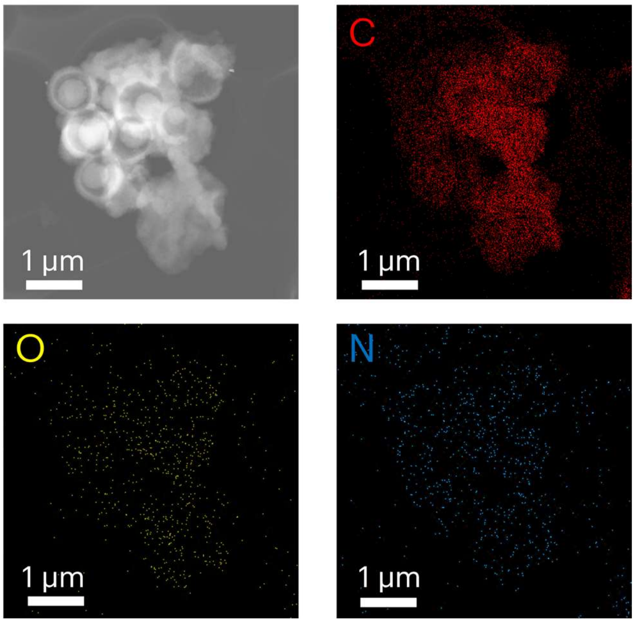

Figure S10. The TEM morphology and elemental distribution maps of COF-Py-CE.

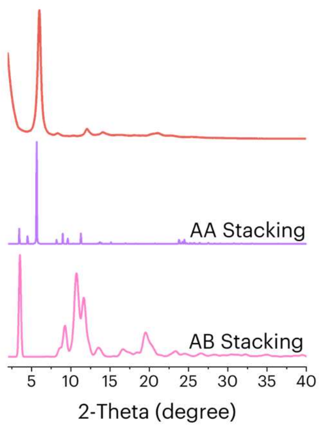

Figure S11. The small-angle XRD pattern of COF-Py-B

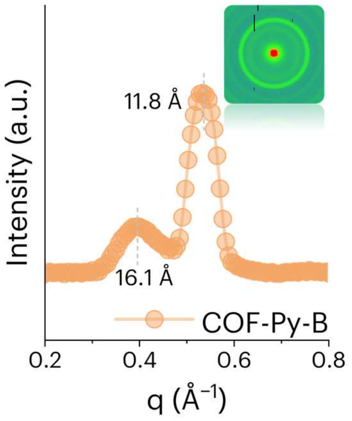

Figure S12. The SAXS pattern of COF-Py-B.

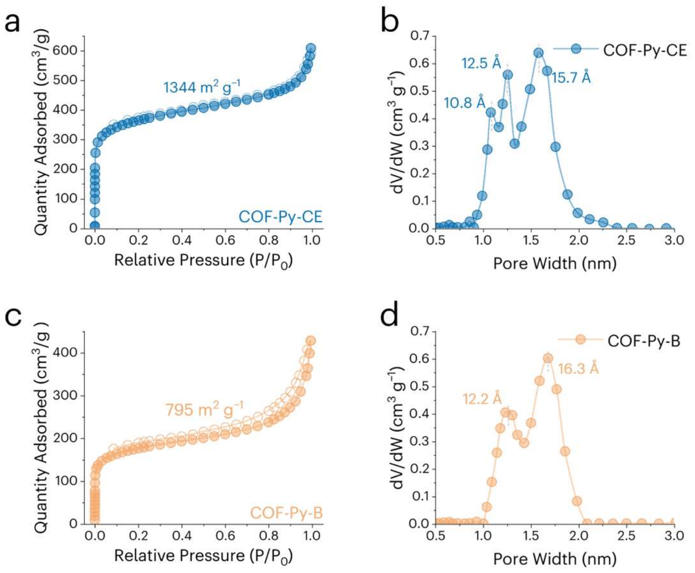

Figure S13. The BET curves of COF-Py-CE and COF-Py-B (a and c), along with their corresponding pore size distribution curves (b and d).

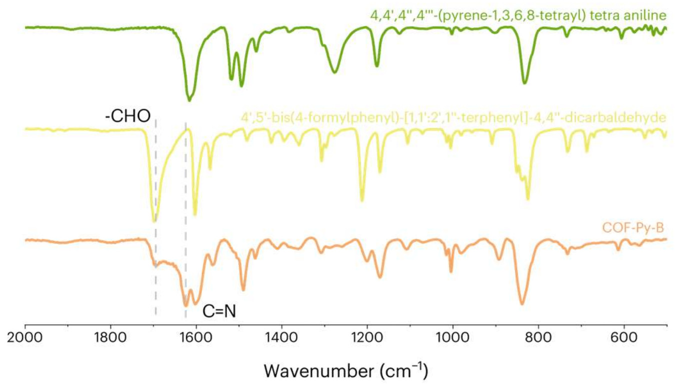

Figure S14. The FTIR spectrum of COF-Py-B.

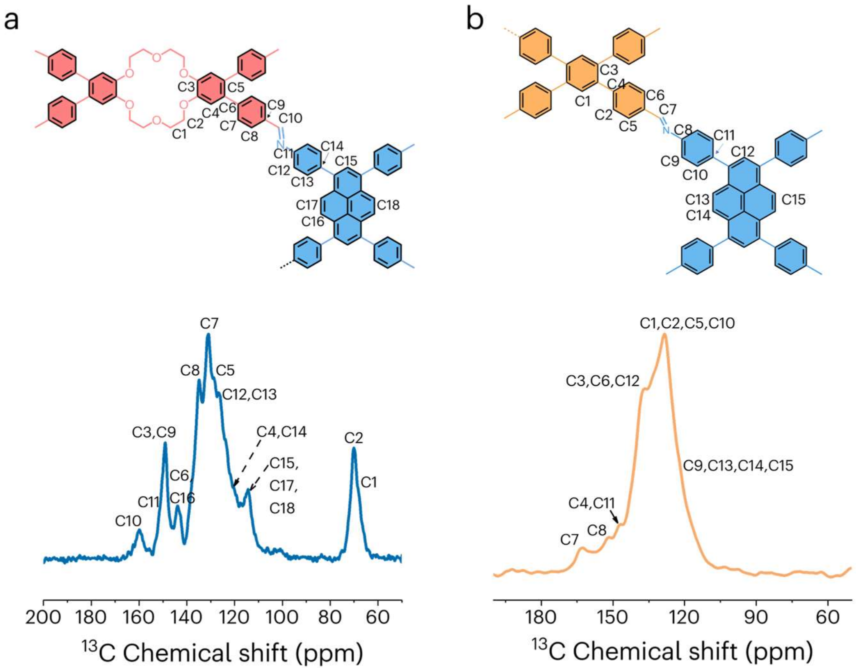

Figure S15. a,b The solid-state ^{13}C NMR spectra of COF-Py-CE and COF-Py-B.

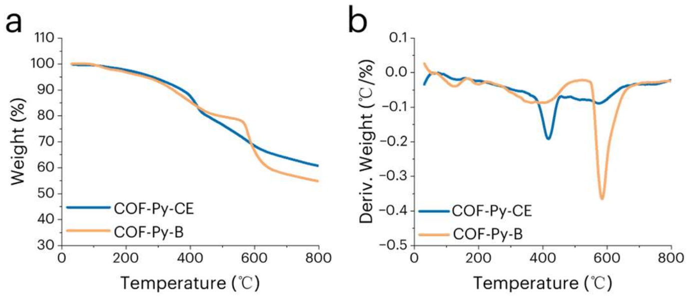

Figure S16. (a) The thermogravimetric (TGA) curves of COF-Py-CE and COF-Py-B. (b) The first-derivative curves of the TGA profiles.

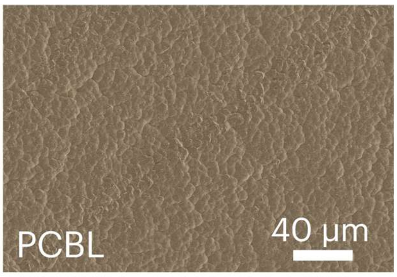

Figure S17. The surface SEM morphology of PCBL.

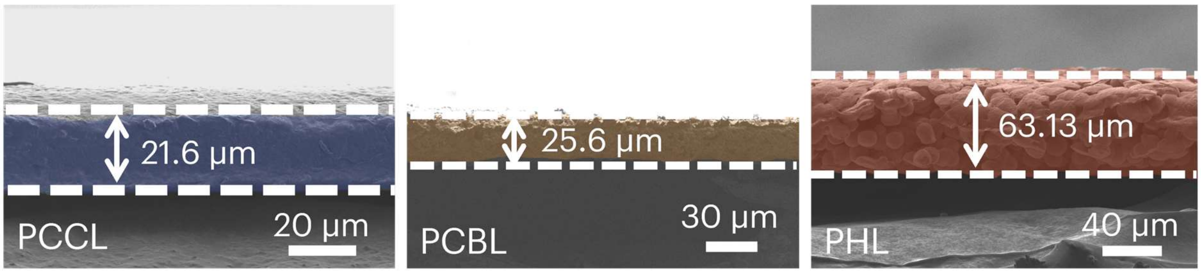

Figure S18. The cross-sectional SEM images of PCCL, PCBL and PHL.

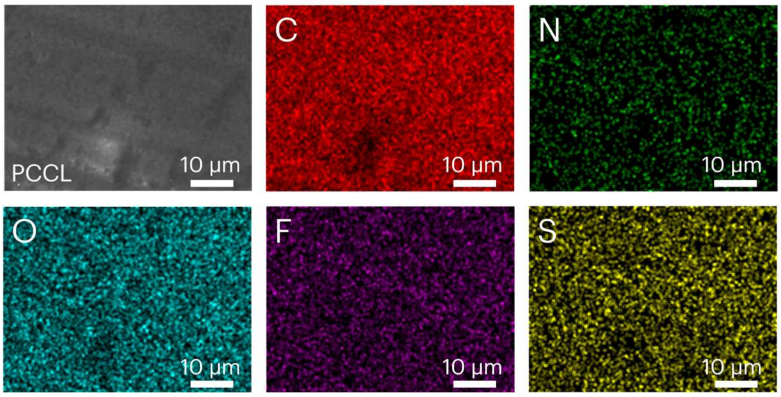

Figure S19. The EDS elemental distribution maps of the PCCL membrane surface.

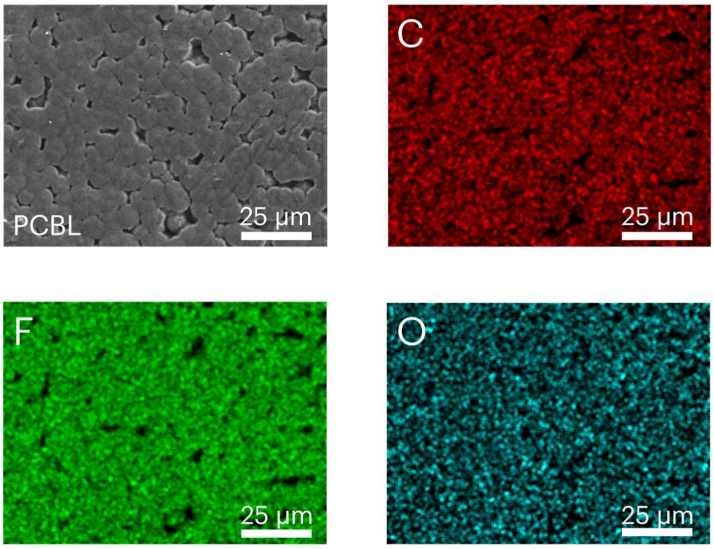

Figure S20. The EDS elemental distribution on the surface of PCBL.

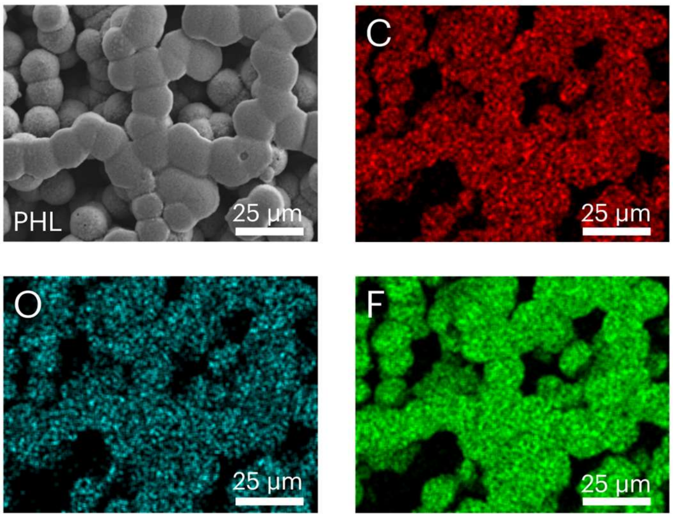

Figure S21. The EDS elemental distribution on the surface of PHL.

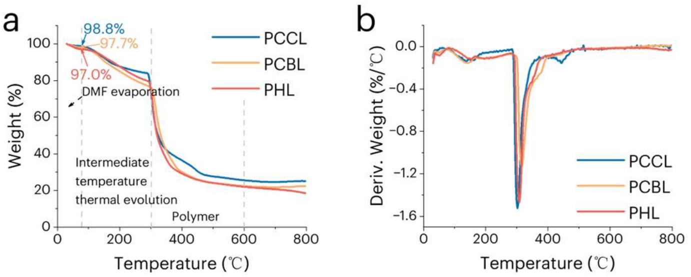

Figure S22. (a) The thermogravimetric (TGA) curve of the electrolyte membrane. (b) The first-derivative curve of the TGA profile.

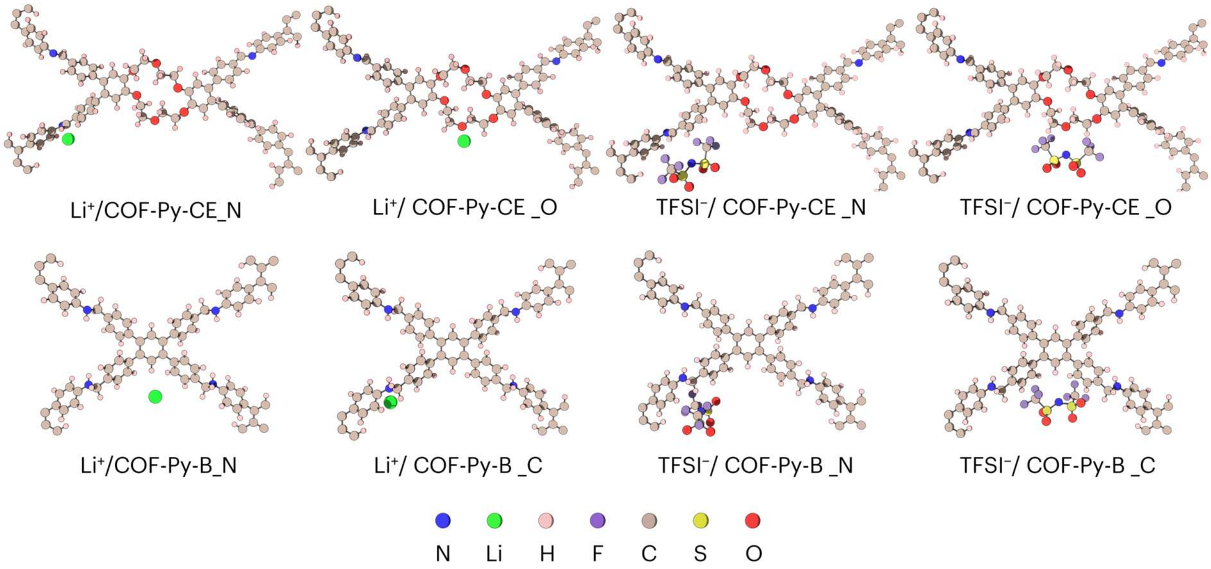

Figure S23. The adsorption models of COF-Py-CE and COF-Py-B for Li^+ and $TFSI^−$ at different sites.

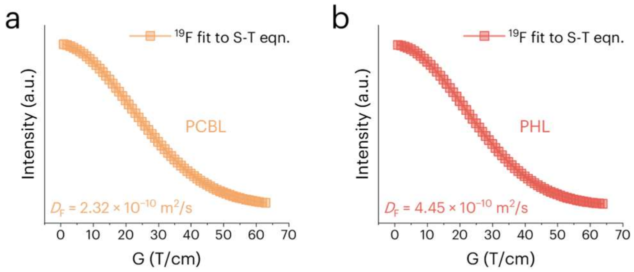

Figure S24. ^{19}F NMR intensity and fitted decay curves used to calculate the diffusion coefficient of $TFSI^-$ anions in the PCBL (a) and PHL (b) electrolyte.

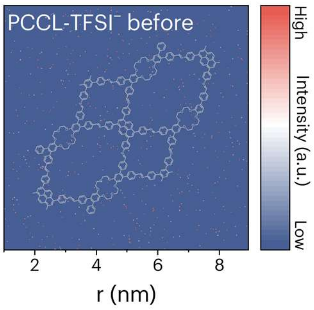

Figure S25. Density distribution map of $TFSI^{-}$ in PCCL before simulation.

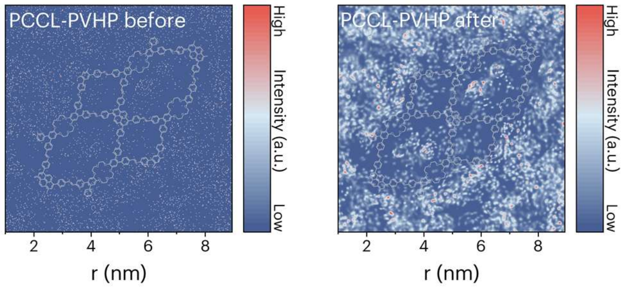

Figure S26. The two-dimensional density distributions of PVDF-HFP molecules in the environment before and after simulation with the incorporation of COF-Py-CE in PCCL.

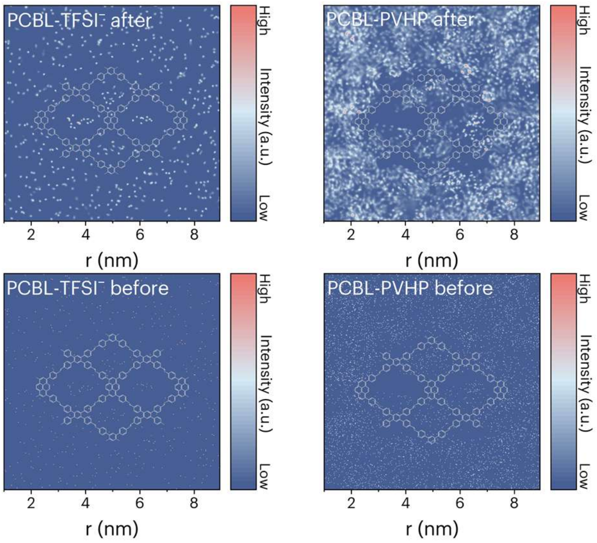

Figure S27. The two-dimensional density distributions of $TFSI^-$ and PVDF-HFP molecules in the environment before and after simulation with the incorporation of COF-Py-B in PCBL.

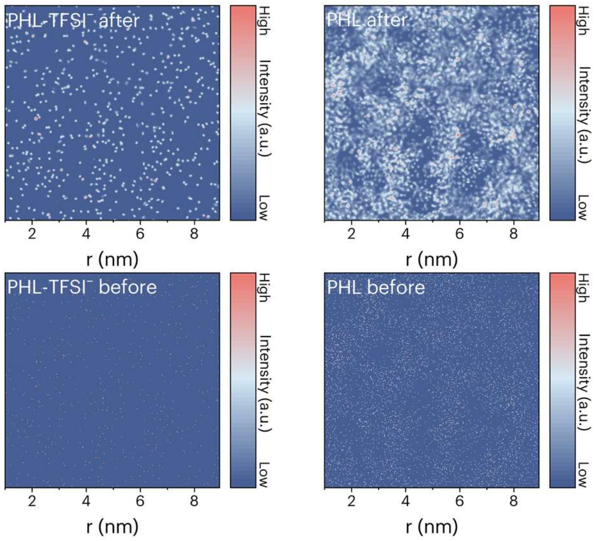

Figure S28. The two-dimensional density distributions of $TFSI^-$ and PVDF-HFP molecules in the environment before and after simulation in the PHL.

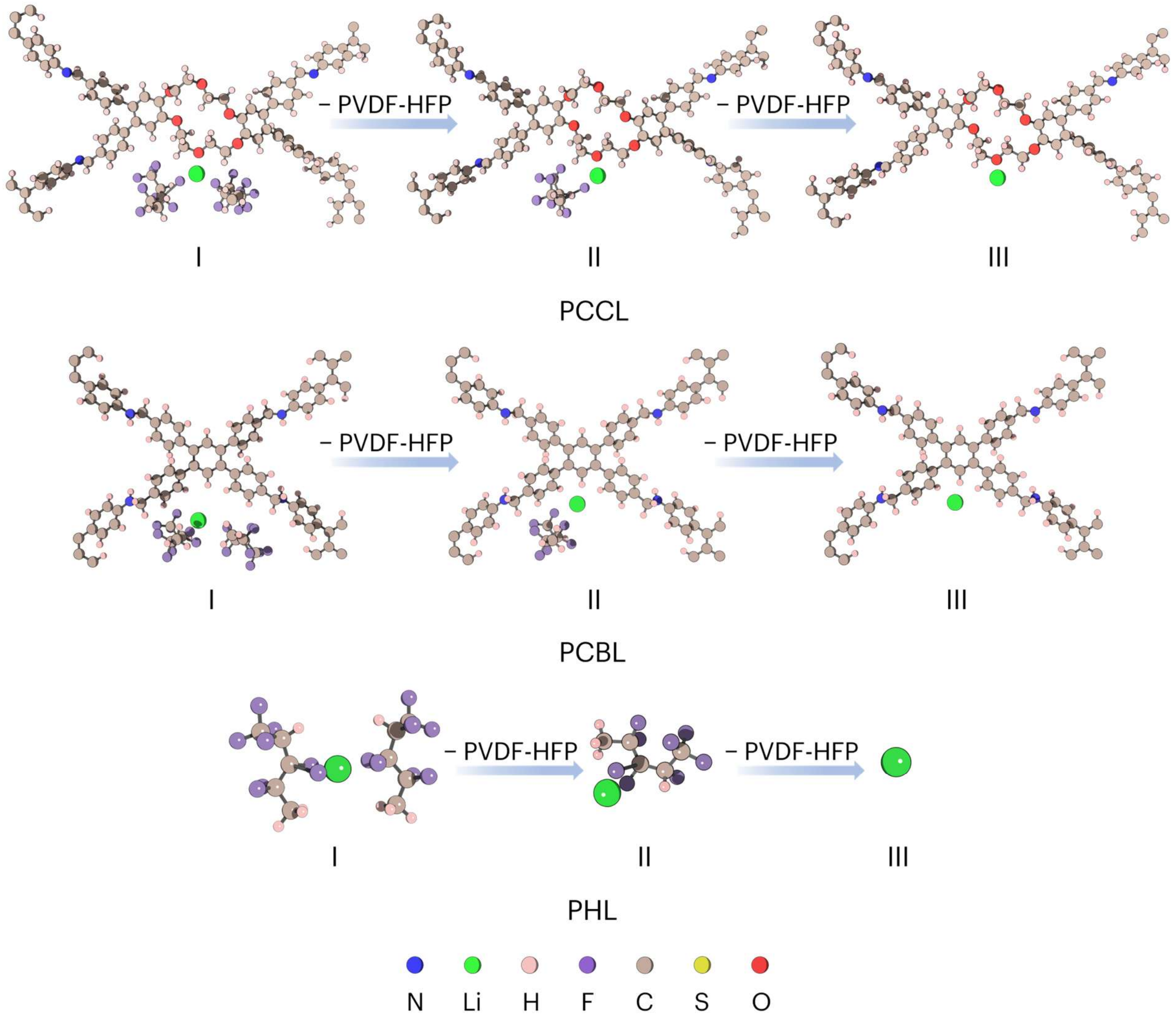

Figure S29. Li^+ migration barrier in soild-state electrolytes. Schematic diagram of the Li^+ transport calculation model in the PCCL, PCBL and PHL electrolyte. The process can be described as follows: Li^+ initially detaches from a PVDF-HFP chain (Process I to II), subsequently detaches from a PVDF-HFP chain (Process II to III), and eventually fully exits the polymer matrix.

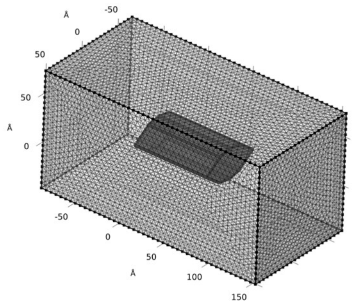

Figure S30. Schematic diagram of the FEM simulation model.

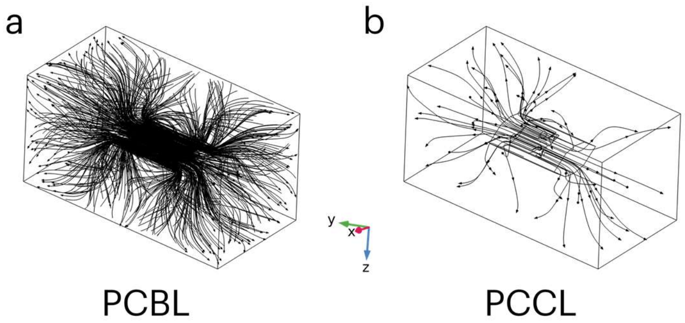

Figure S31. Schematic illustration of FEM-simulated Li^+ outward diffusion into the polymer matrix in PCBL (a) and PCCL (b).

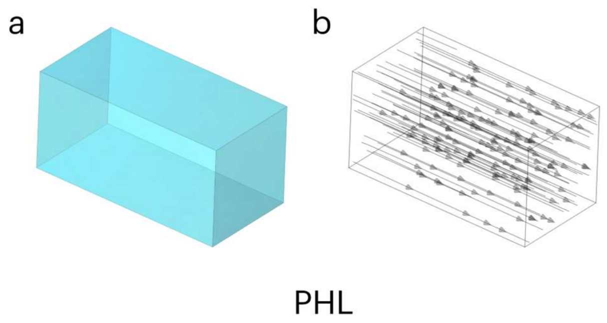

Figure S32. The FEM modeling model (a) and schematic illustration (b) of Li^+ diffusion in PHL.

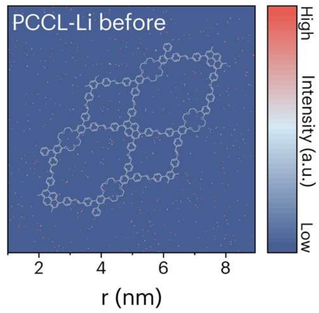

Figure S33. Density distribution map of Li^+ in PCCL before simulation.

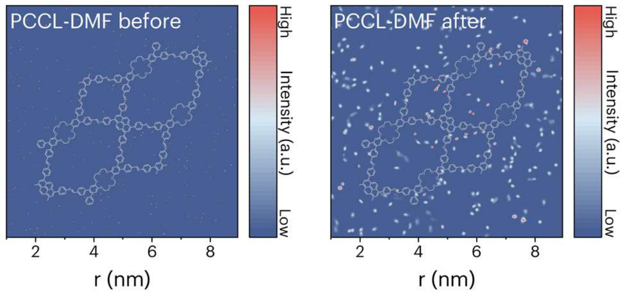

Figure S34. The two-dimensional density distributions of DMF molecules in the environment before and after simulation with the incorporation of COF-Py-CE in PCCL.

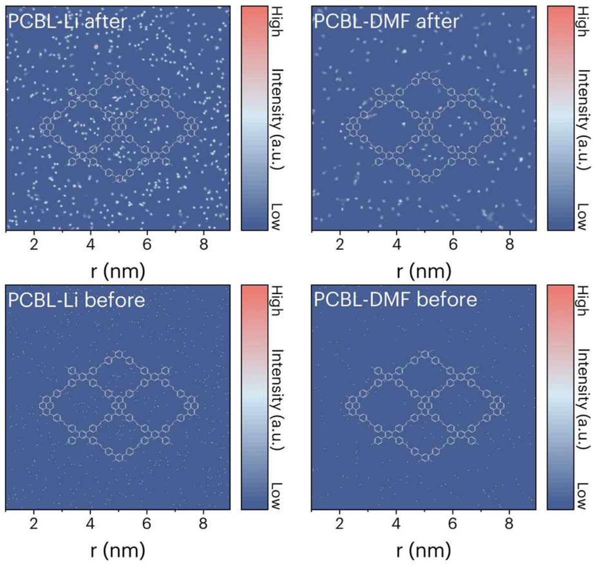

Figure S35. The two-dimensional density distributions of Li^{+} and DMF molecules in the environment before and after simulation with the incorporation of COF-Py-B in PCBL.

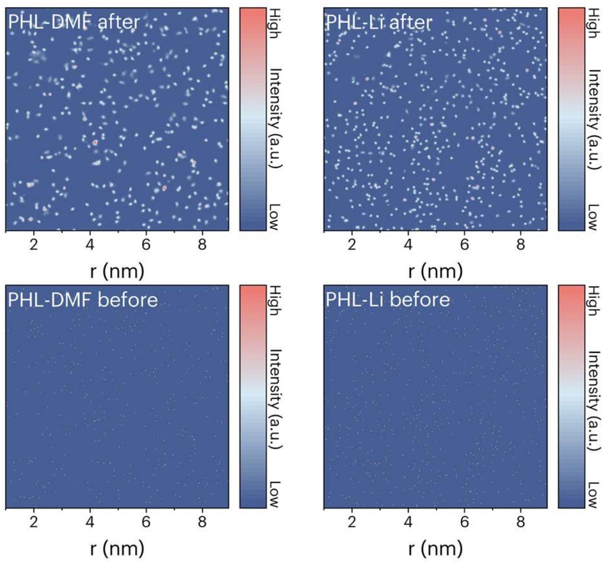

Figure S36. The two-dimensional density distributions of Li^+ and DMF molecules in the environment before and after simulation in PHL.

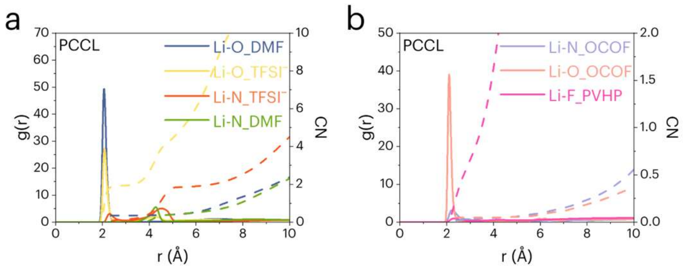

Figure S37. Radial distribution function (RDF) curves and coordination numbers for the PCCL electrolyte.

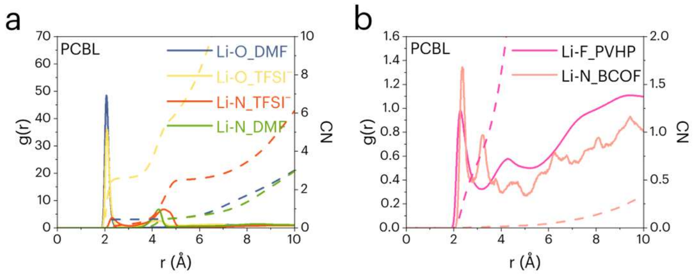

Figure S38. Radial distribution function (RDF) curves and coordination numbers for the PCBL electrolyte.

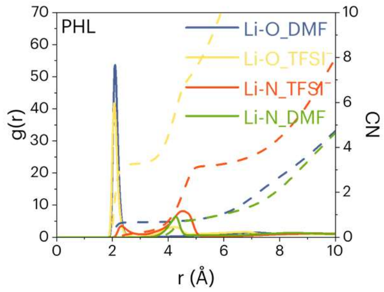

Figure S39. Radial distribution function (RDF) curves and coordination numbers for the PHL electrolyte.

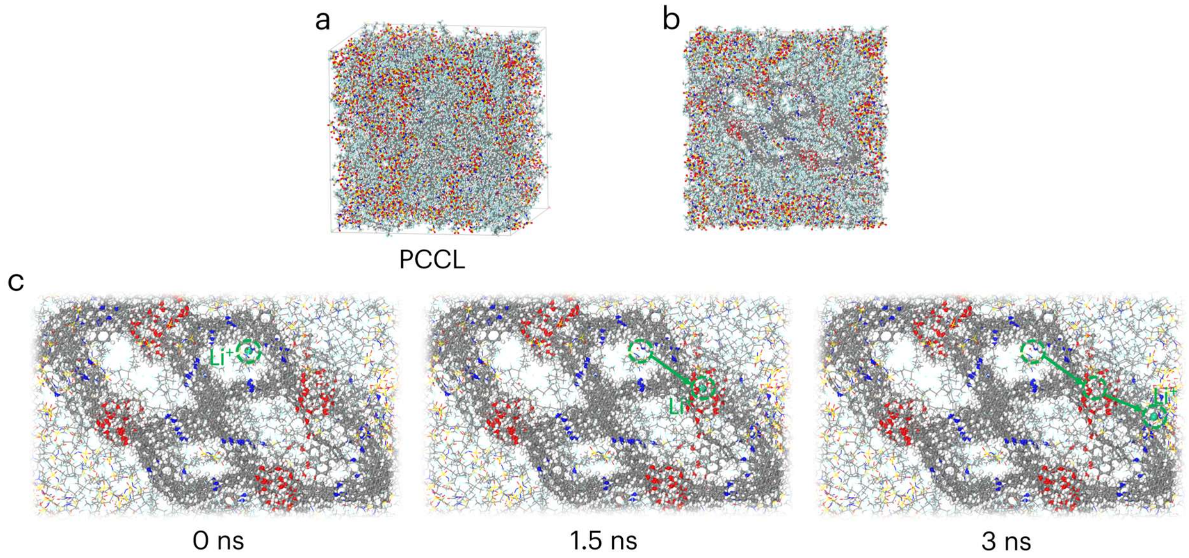

Figure S40. Molecular dynamics simulation of the PCCL solid-state electrolyte. (a, b) Molecular models of PCCL. (c) MD simulation snapshot of Li^+ migration along the in-plane direction of COF-Py-CE within PCCL.

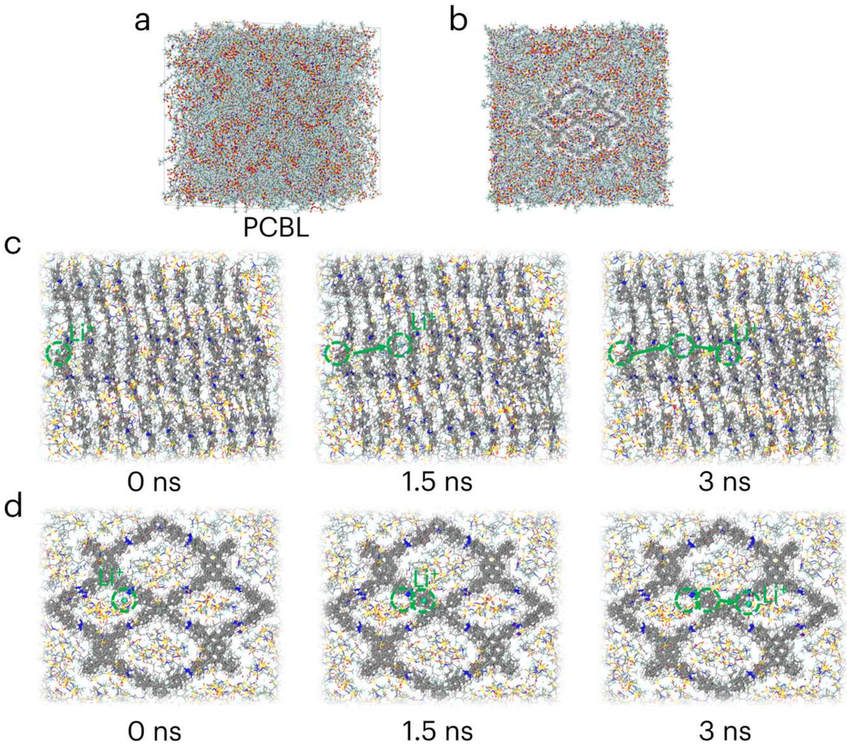

Figure S41. Molecular dynamics simulation of the PCBL solid-state electrolyte. (a, b) Molecular models of PCBL. (c) MD simulation snapshot of Li^{+} hopping migration across the interlayer spacing of COF-Py-B in PCBL. (d) MD simulation snapshot of Li^{+} migration along the in-plane direction of COF-Py-B within PCBL.

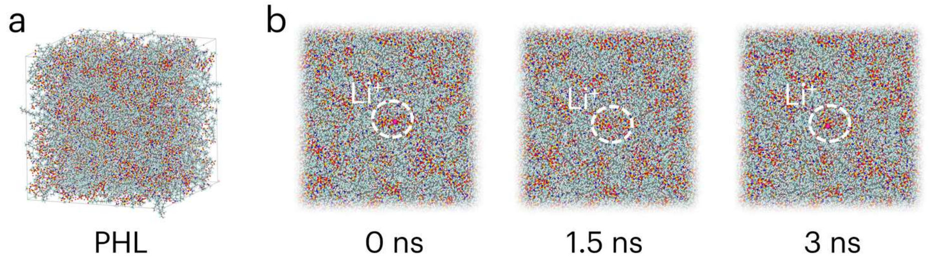

Figure S42. Molecular dynamics simulation of PHL solid electrolytes. (a) Molecular model of PHL. (b) MD simulation snapshots of migrating Li^+ in PHL.

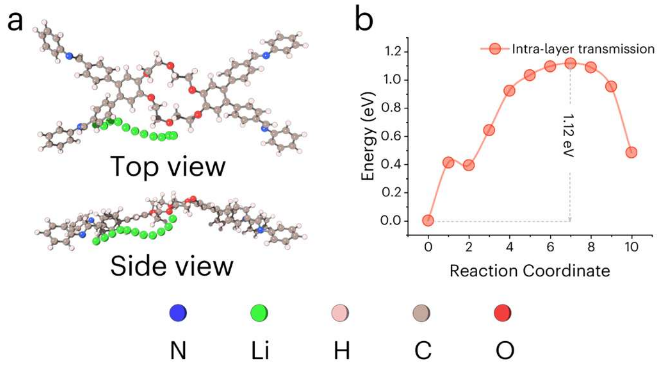

Figure S43. (a) Schematic model of Li^+ intralayer migration in COF-Py-CE (N→O). (b) Energy values at each point during Li^+ intralayer migration (N→O).

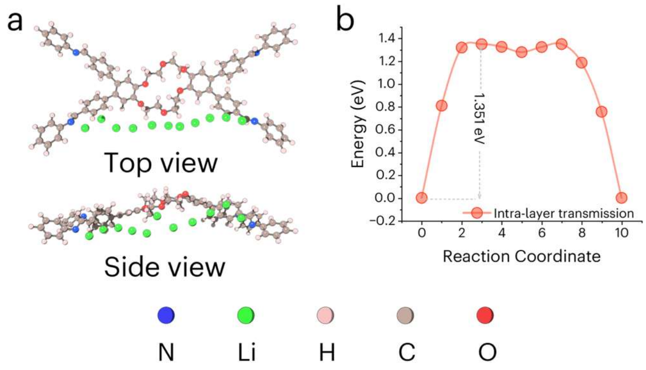

Figure S44. (a) Schematic model of Li^+ intralayer migration in COF-Py-CE (N→N). (b) Energy values at each point during Li^+ intralayer migration (N→N).

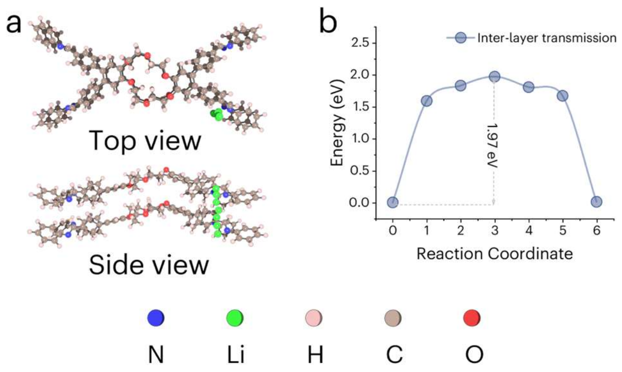

Figure S45. (a) Schematic model of Li^+ interlayer migration in COF-Py-CE (N→N). (b) Energy values at each point during Li^+ interlayer migration (N→N).

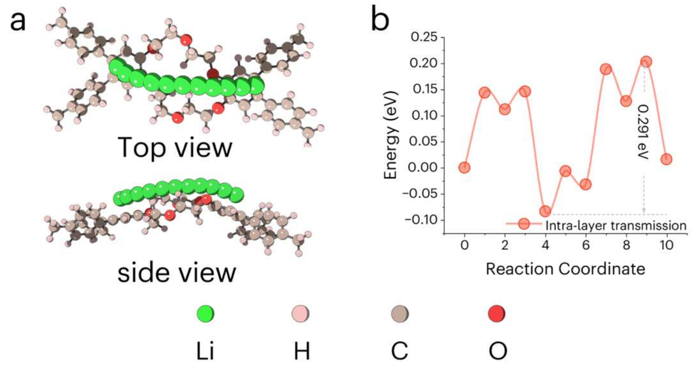

Figure S46. Transition-state simulation of Li^+ in-plane migration pathways within COF-Py-CE. (a) Schematic model of Li^+ in-plane migration. (b) Energy profile corresponding to each position along the migration pathway.

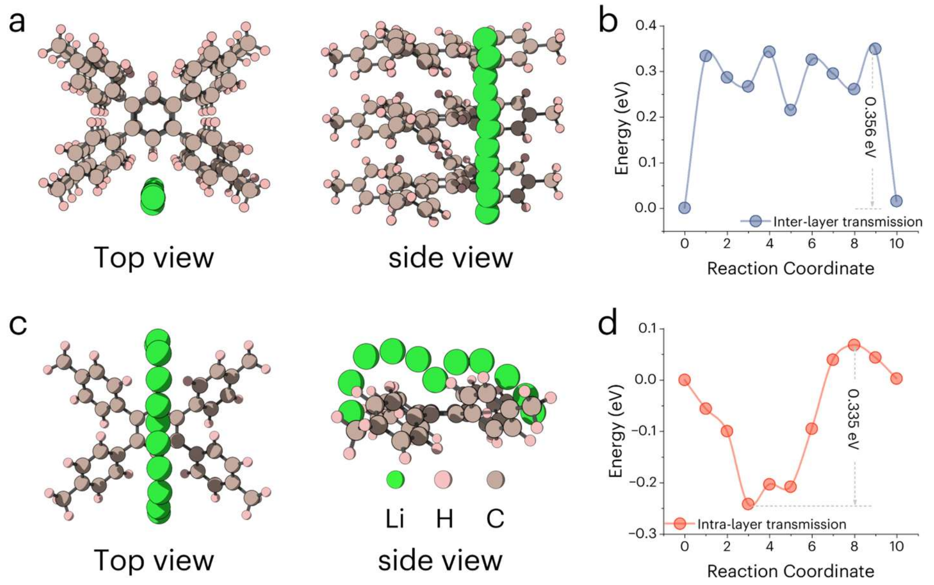

Figure S47. Transition-state simulations of Li^+ migration pathways in COF-Py-B. (a) Schematic model of Li^+ interlayer migration. (b) Energy profile corresponding to each position along the interlayer migration pathway. (c) Schematic model of Li^+ in-plane migration. (d) Energy profile corresponding to each position along the in-plane migration pathway.

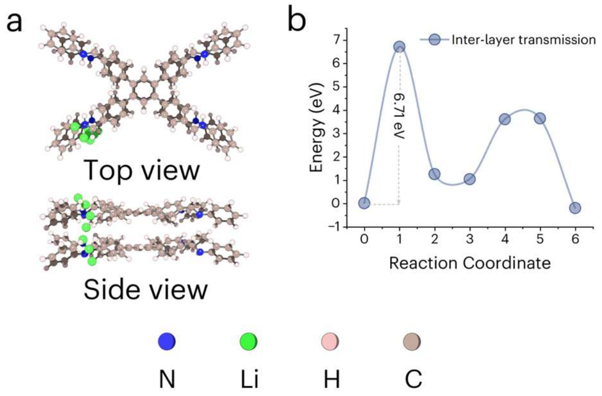

Figure S48. (a) Schematic model of Li^+ interlayer migration in COF-Py-B (N→N). (b) Energy values at each point during Li^+ interlayer migration (N→N).

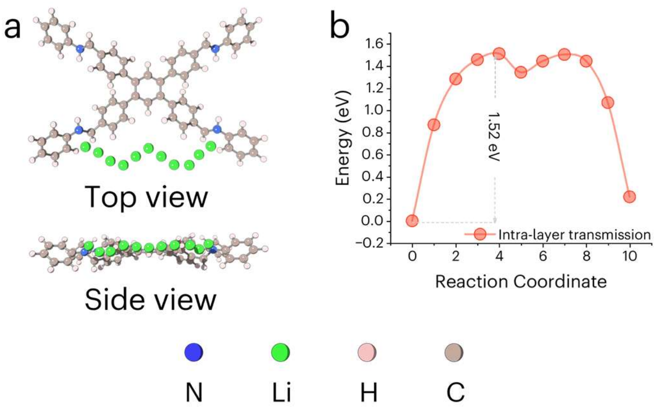

Figure S49. (a) Schematic model of Li^+ intralayer migration in COF-Py-B (N→N). (b) Energy values at each point during Li^+ intralayer migration (N→N).

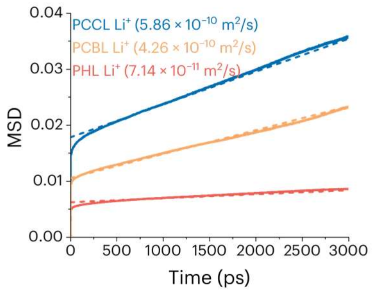

Figure S50. MSD curves of Li^+ in the PCCL, PCBL, and PHL electrolyte.

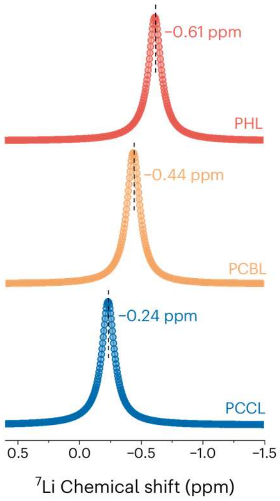

Figure S51. ^{7}Li solid-state NMR spectra of PCCL, PCBL and PHL.

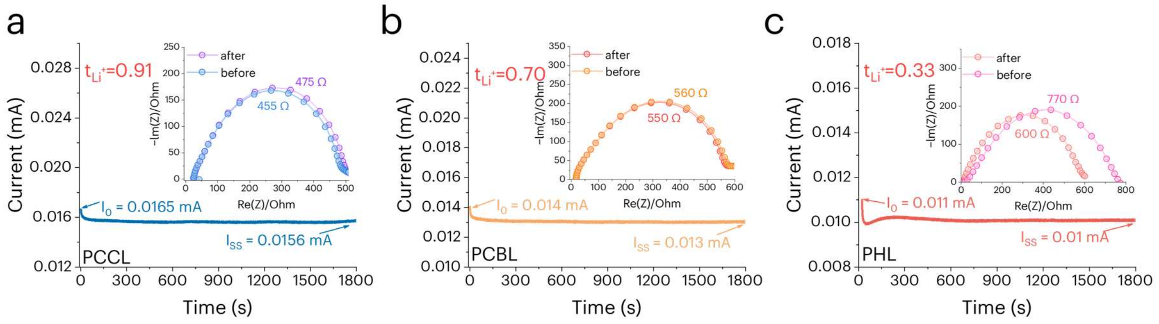

Figure S52. Current-time curves for symmetric cells. (a) Li/PCCL/Li, (b) Li/PCBL/Li and (c) Li/PHL/Li under a 10 mV DC voltage. Insets show the EIS before and after polarization.

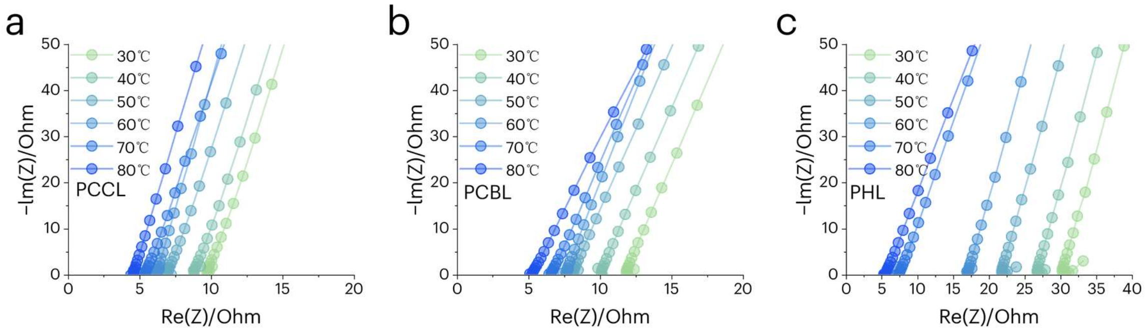

Figure S53. Arrhenius plots. (a) PCCL, (b) PCBL and (c) PHL.

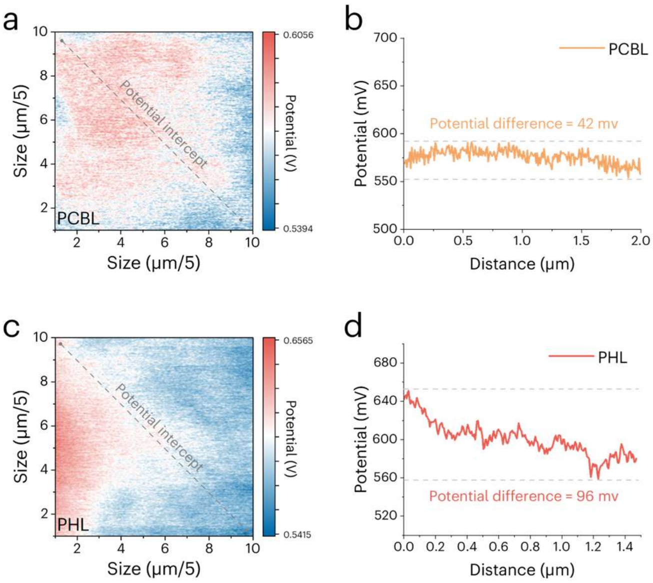

Figure S54. KPFM potential distribution images of the PCBL (a) and PHL (b) electrolyte membranes. The potential differences within the PCBL (c) and PHL (d) membranes.

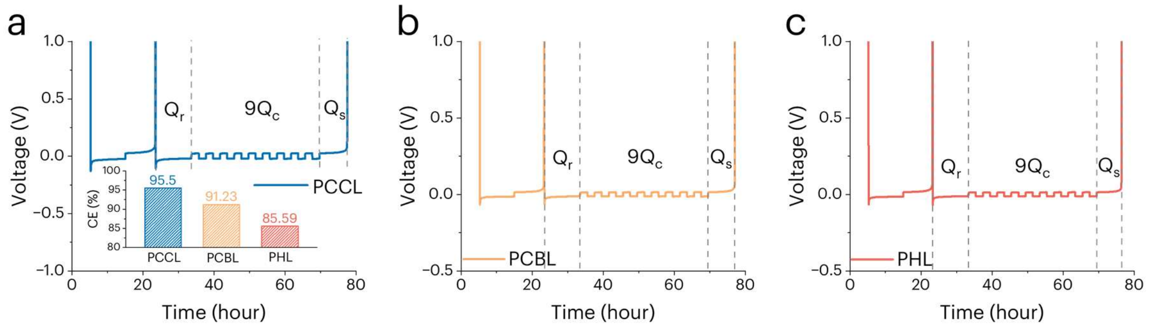

Figure S55. Coulombic efficiency and cycling performance of Li||Cu cells using PCCL, PCBL and PHL electrolytes.

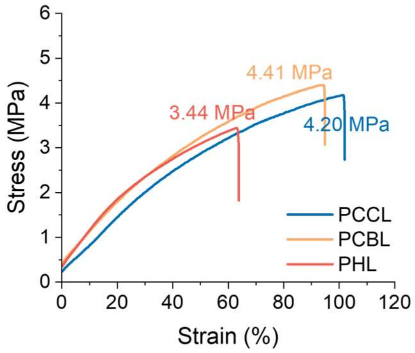

Figure S56. Fracture elongation curve of the electrolyte membranes.

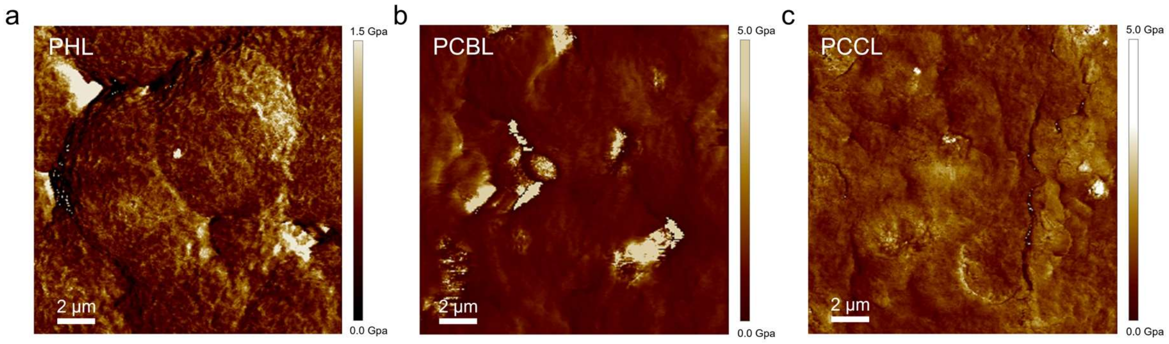

Figure S57. (a) PHL, (b) PCBL, and (c) PCCL Young's modulus distribution maps.

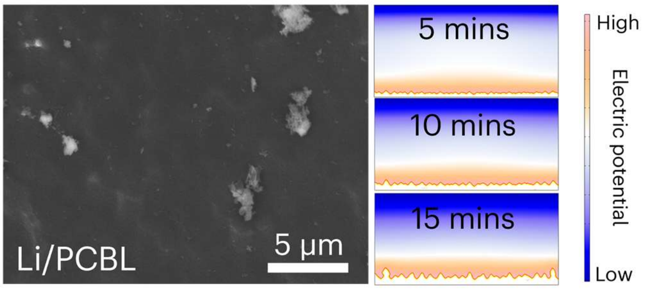

Figure S58. SEM image of the Li metal anode surface morphology with PCBL electrolyte. FEM simulation of dendrite growth and current density variations on the lithium metal anode surface using PCBL electrolyte.

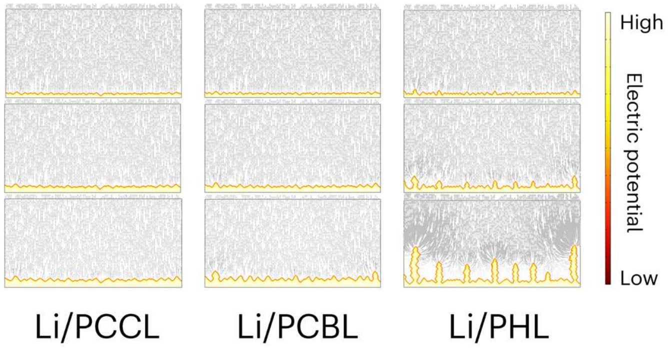

Figure S59. Finite element simulations of dendrite growth and its evolution on the lithium metal anode surface using PCCL, PCBL, and PHL electrolytes.

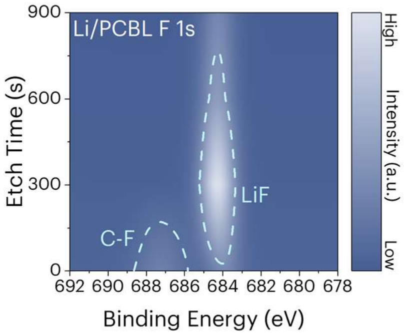

Figure S60. Two-dimensional XPS analysis of F 1s on the surface and depth profile of Li/PCBL as a function of etching time.

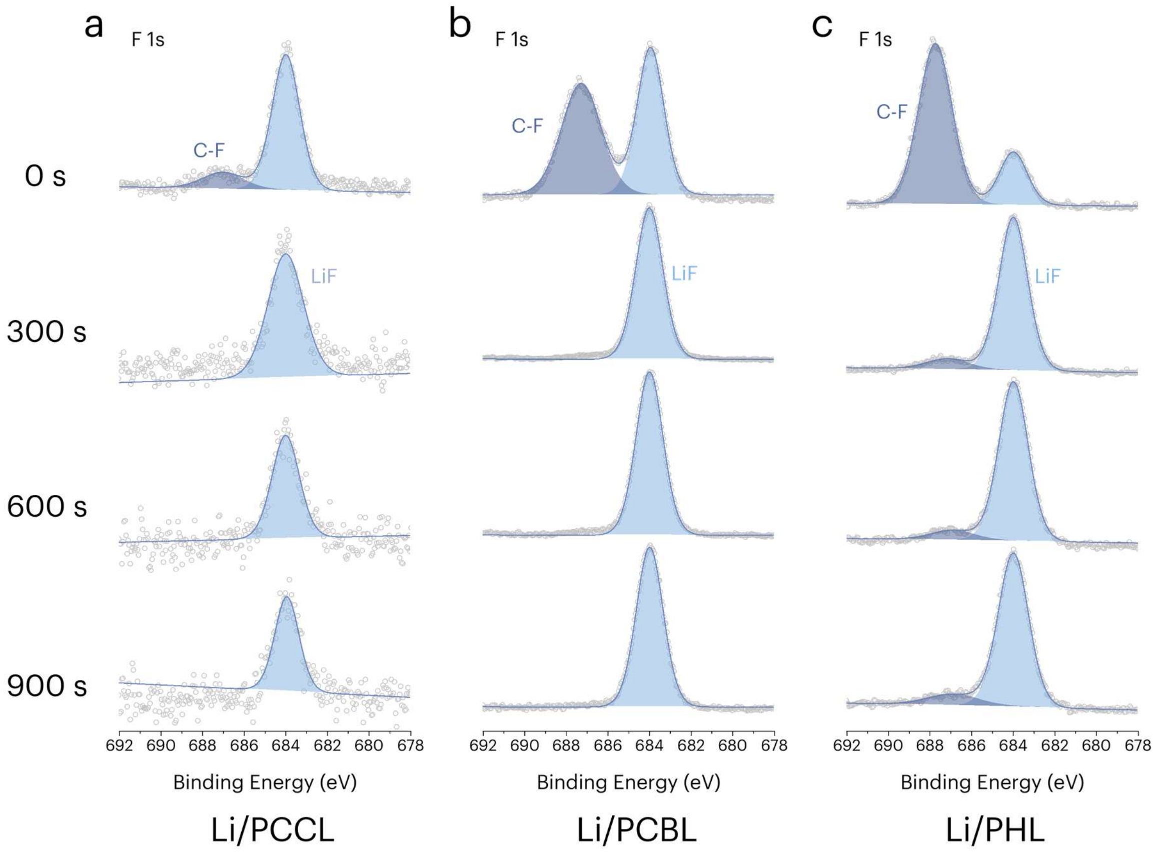

Figure S61. High-resolution XPS fitting curves of F 1s on the surface and depth profile. (a) Li/PCCL, (b) Li/PCBL and (c) Li/PHL.

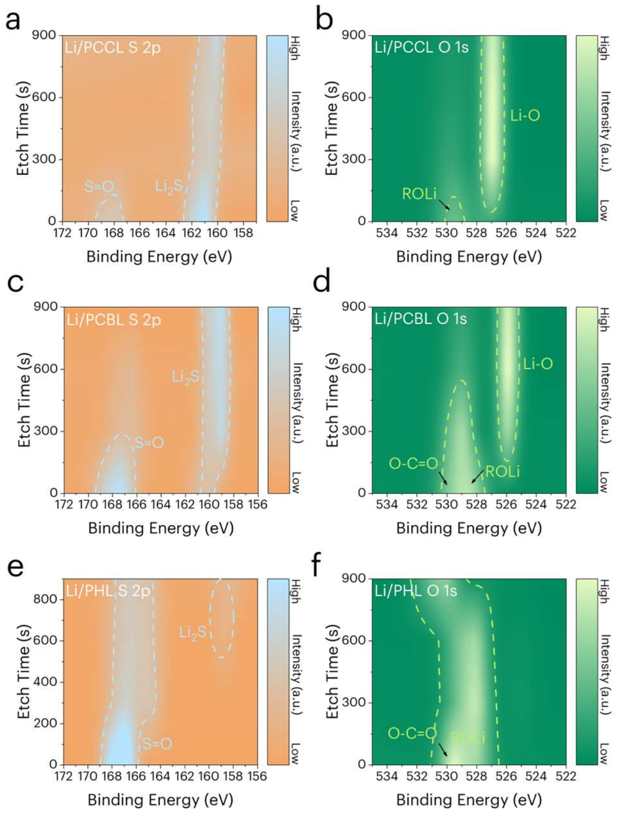

Figure S62. Two-dimensional XPS analysis of S 2p and O 1s on the surface and depth profile of Li/PCCL, Li/PCBL and Li/PHL as a function of etching time.

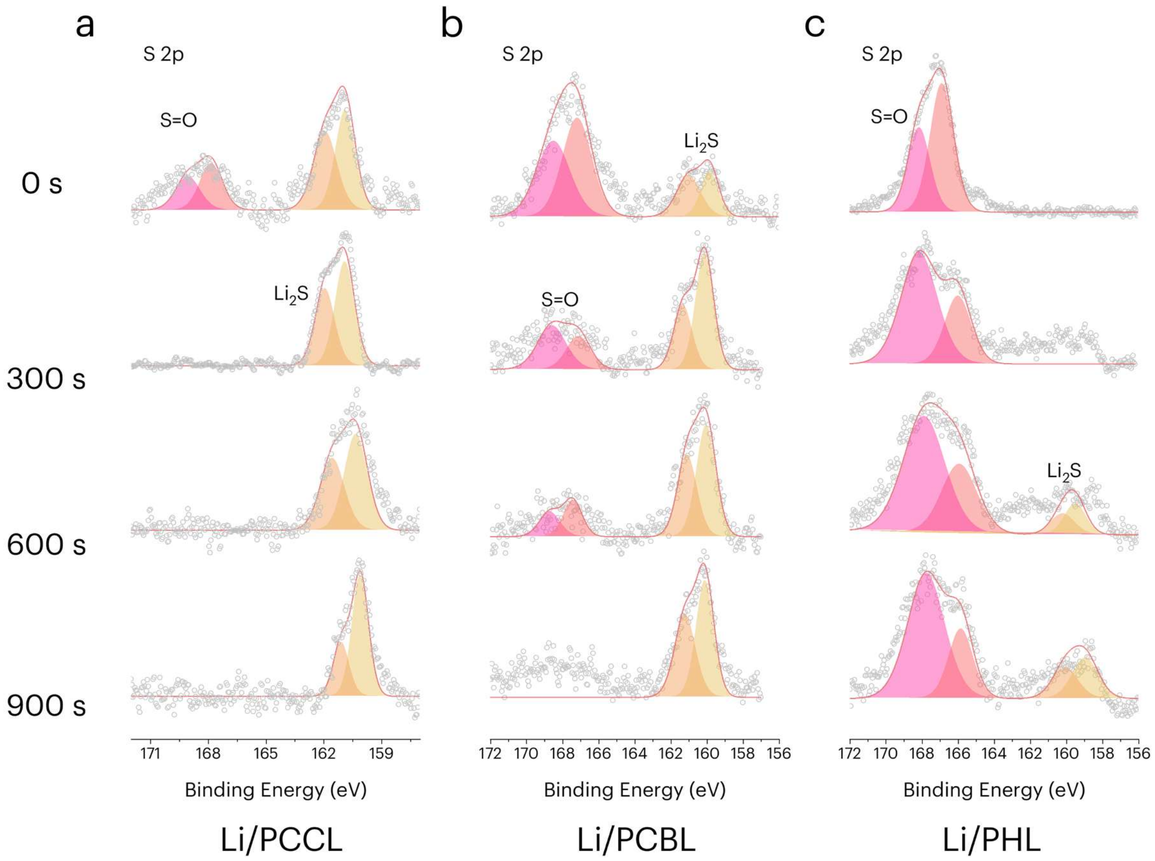

Figure S63. High-resolution XPS fitting curves of S 2p on the surface and depth profile. (a) Li/PCCL, (b) Li/PCBL and (c) Li/PHL.

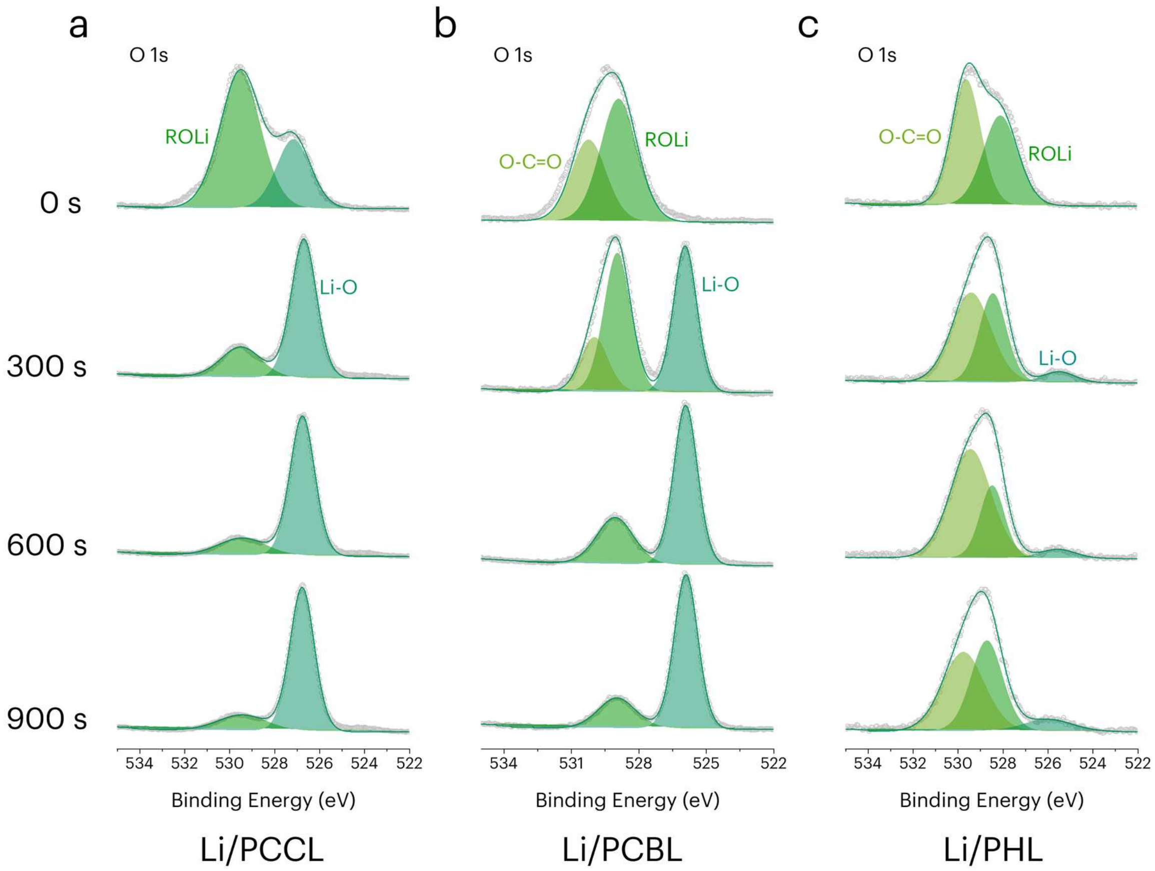

Figure S64. High-resolution XPS fitting curves of O 1s on the surface and depth profile. (a) Li/PCCL, (b) Li/PCBL and (c) Li/PHL.

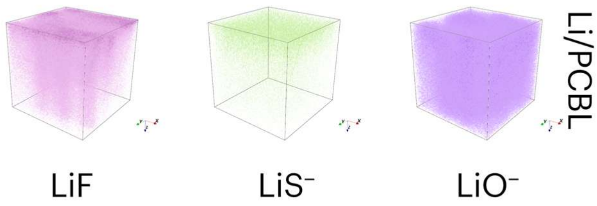

Figure S65. TOF-SIMS 3D reconstruction of representative species forming the SEI layer on Li/PCBL.

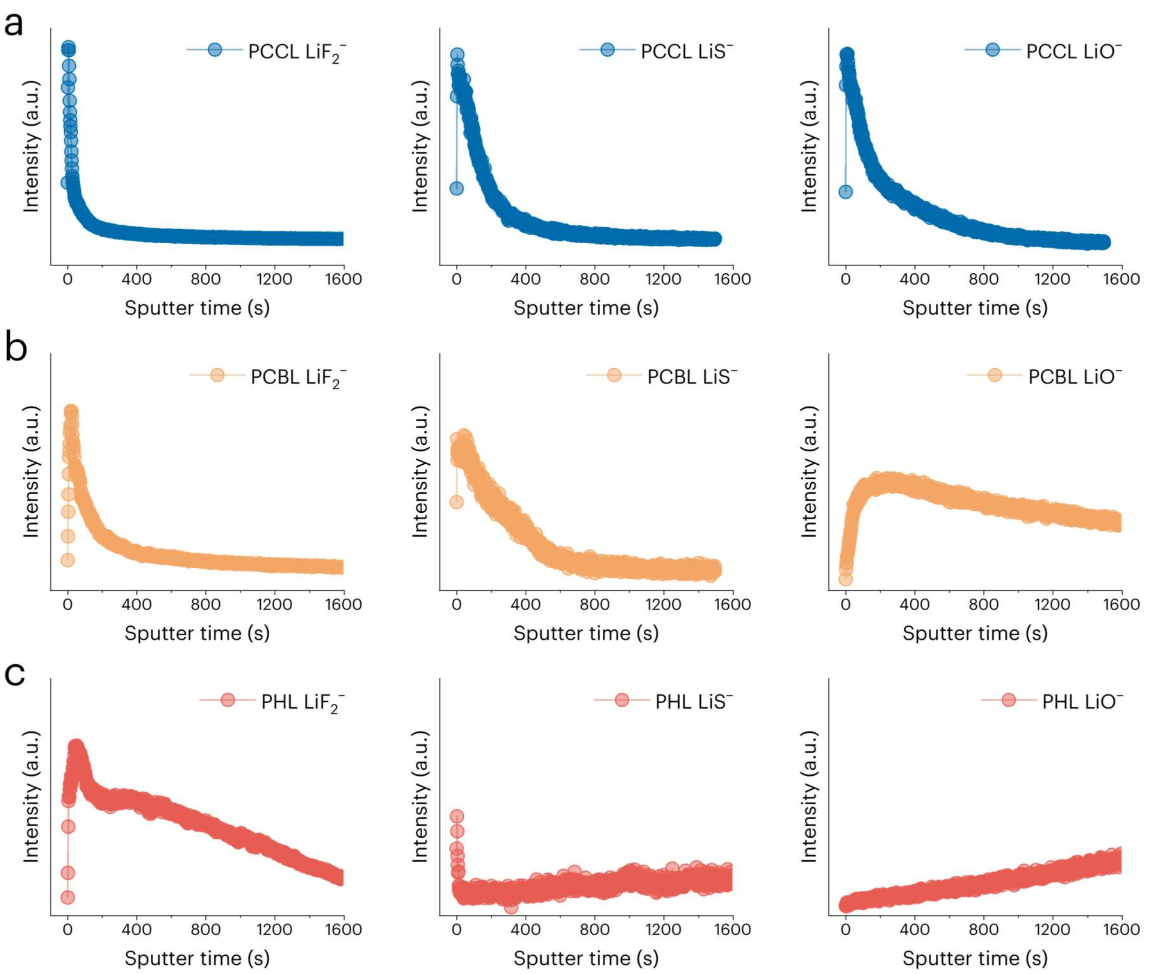

Figure S66. TOF-SIMS curves of LiF_2^-, LiS^- and LiO^-. (a) Li/PCCL, (b) Li/PCBL and (c) Li/PHL.

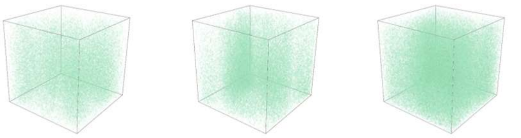

Figure S67. TOF-SIMS 3D reconstruction of NiO_2^- on Cathode/PCCL, Cathode/PCBL and Cathode/PHL.

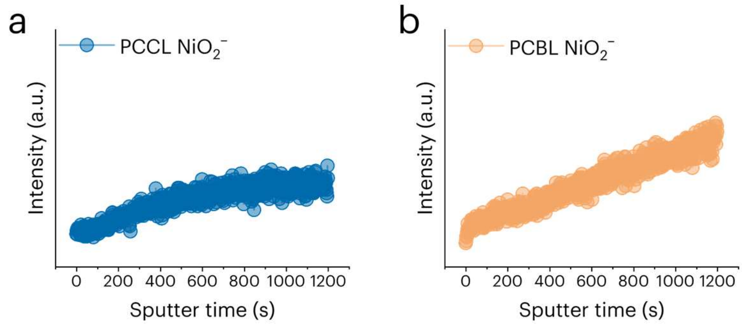

Figure S68. TOF-SIMS curves of NiO_2^-. (a) Cathode/PCCL, (b) Cathode/PCBL and (c) Cathode/PHL.

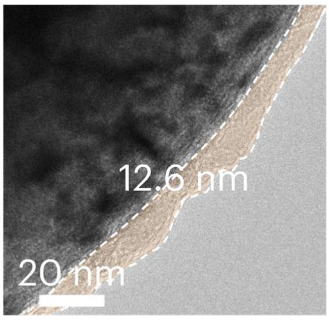

Figure S69. TEM image of the CEI layer at the cathode/PCBL interface.

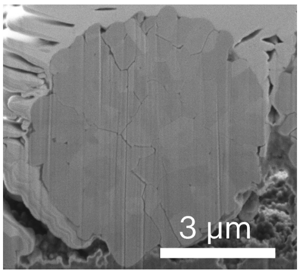

Figure S70. FIB cross-sectional morphology of the NCM523 cathode after cycling with the PCBL electrolyte.

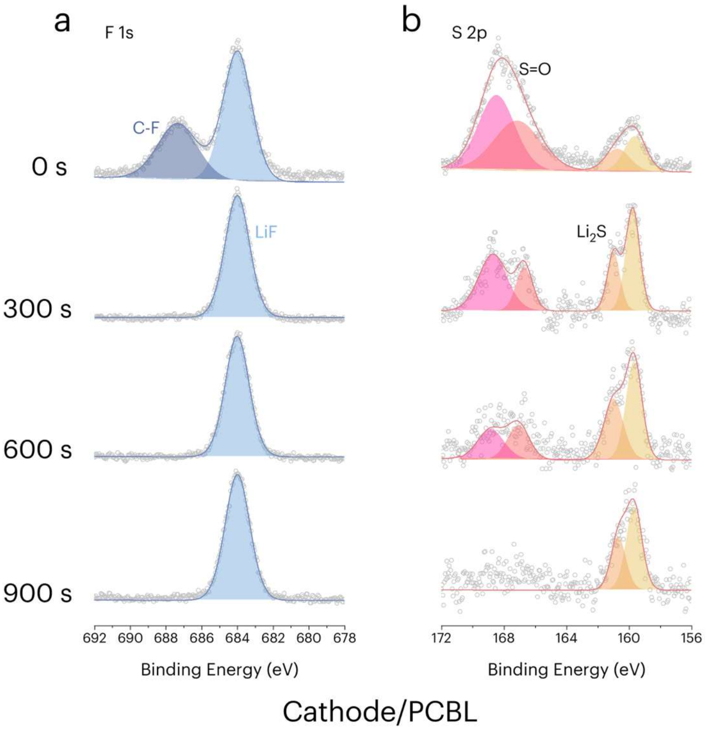

Figure S71. High-resolution XPS fitting curves of F 1s and S 2p on the surface and depth profile of the cathode CEI layer.

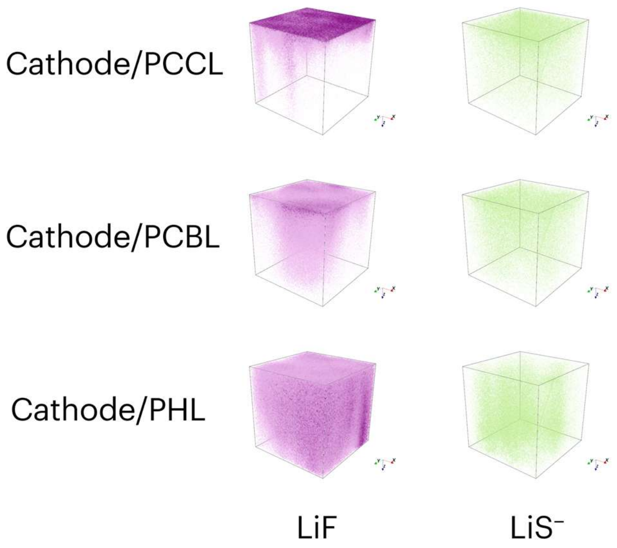

Figure S72. TOF-SIMS 3D reconstruction of representative species forming the CEI layer on Cathode/PCCL, Cathode/PCBL and Cathode/PHL.

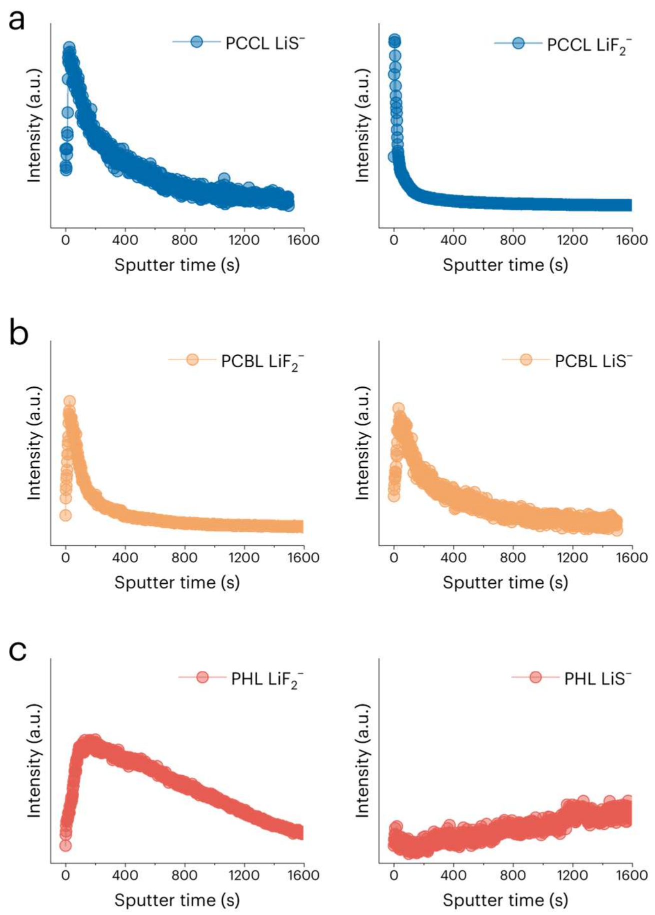

Figure S73. TOF-SIMS curves of LiF_2^- and LiS^- (a) Cathode/PCCL. (b)Cathode/PCBL. (c) Cathode/PHL.

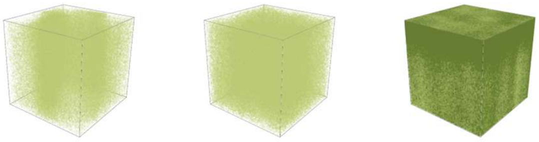

Figure S74. TOF-SIMS 3D reconstruction of NiO_3^- on Cathode/PCCL, Cathode/PCBL and Cathode/PHL.

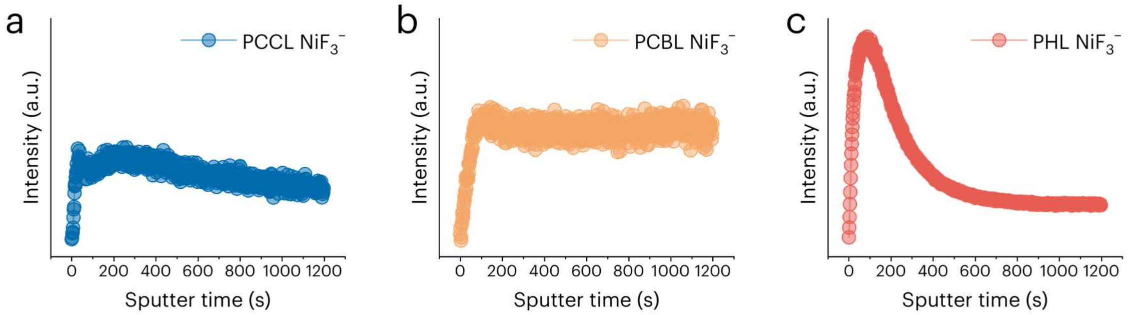

Figure S75. TOF-SIMS curves of NiF_3^-. (a) Cathode/PCCL, (b) Cathode/PCBL and (c) Cathode/PHL.

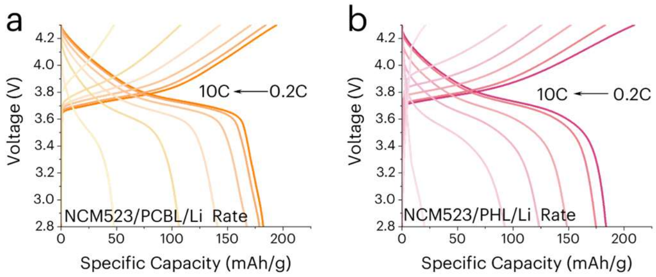

Figure S76. Charge-discharge curves of NCM523/Li batteries using different electrolytes at various rates. (a) PCBL and (b) PHL.

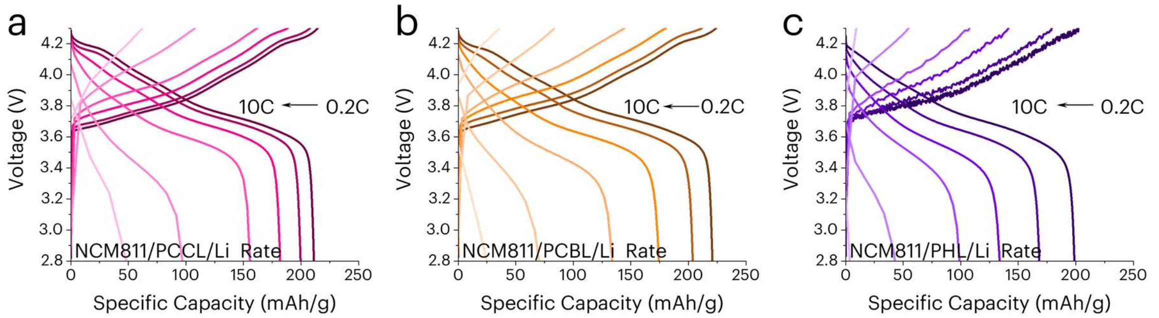

Figure S77. Charge-discharge curves of NCM811/Li batteries using different electrolytes at various rates. (a) PCCL, (b) PCBL and (c) PHL.

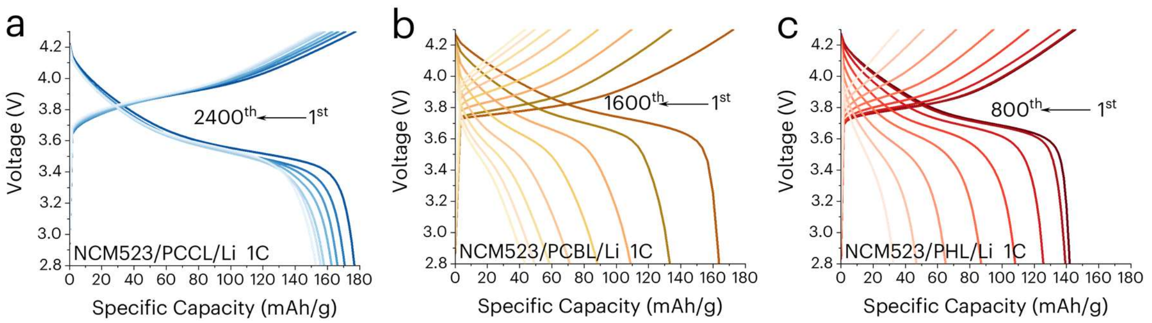

Figure S78. Charge-discharge curves of NCM523/Li batteries using different electrolytes during 1 C cycling. (a) PCCL, (b) PCBL and (c) PHL.

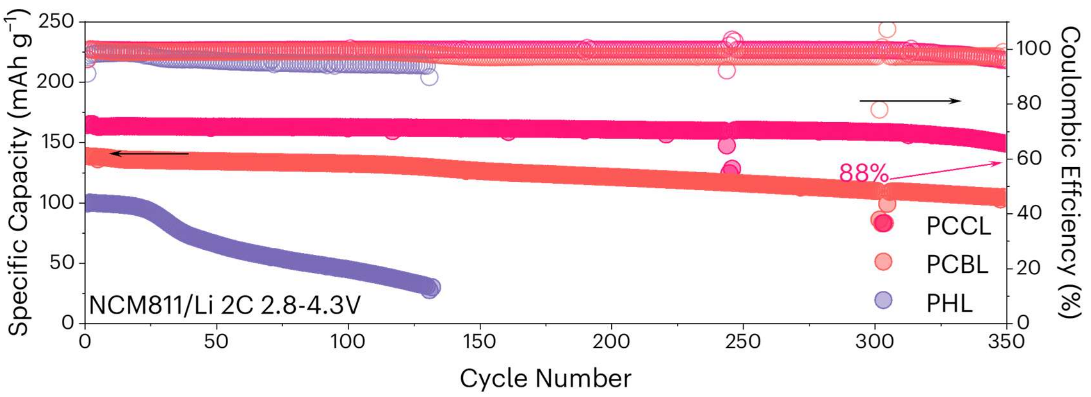

Figure S79. Long-term cycling stability of NCM811/Li batteries using different electrolytes at 2 C and RT.

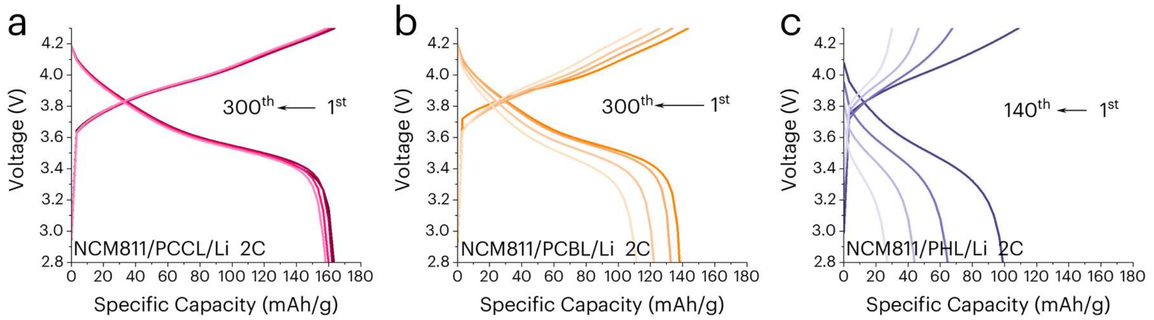

Figure S80. Charge-discharge curves of NCM811/Li batteries using different electrolytes during 2 C cycling. (a) PCCL, (b) PCBL and (c) PHL.

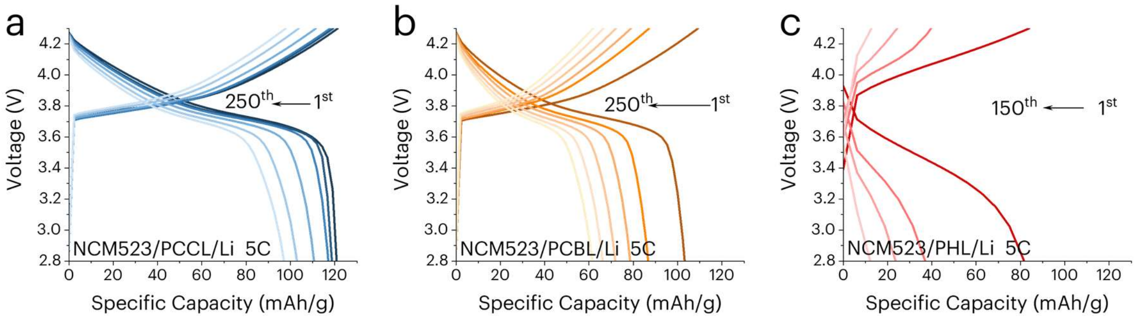

Figure S81. Charge-discharge curves of NCM523/Li batteries using different electrolytes during 5 C cycling. (a) PCCL, (b) PCBL and (c) PHL.

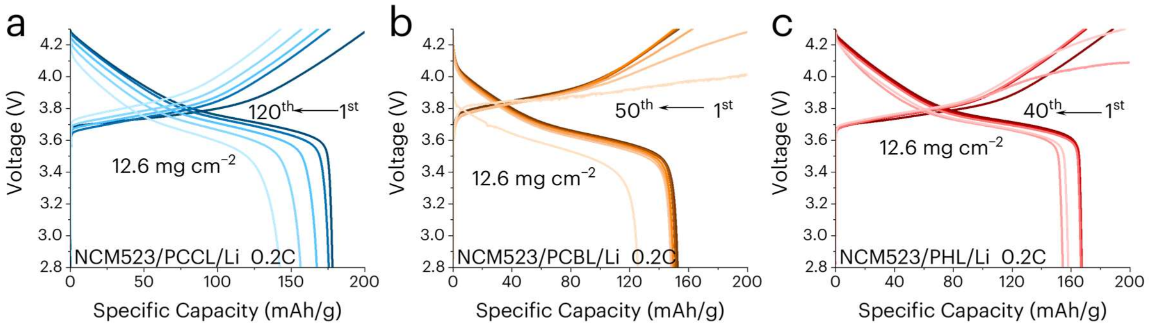

Figure S82. Charge-discharge curves of NCM523/Li batteries under high cathode loading conditions. (a) PCCL, (b) PCBL and (c) PHL.

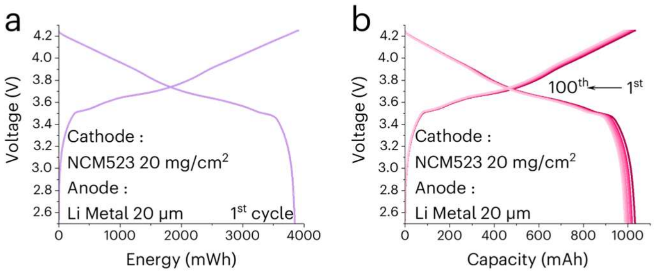

Figure S83. The initial energy of NCM523/PCCL/Li pouch cell.

References

(1) Grimme, S.; Antony, J.; Ehrlich, S.; Krieg, H. A Consistent and Accurate *Ab Initio* Parametrization of Density Functional Dispersion Correction (DFT-D) for the 94 Elements H-Pu. *J. Chem. Phys.* **2010**, *132* (15), 154104.

(2) Guan, D.; Xu, H.; Zhang, Q.; Huang, Y.-C.; Shi, C.; Chang, Y.-C.; Xu, X.; Tang, J.; Gu, Y.; Pao, C.-W.; Haw, S.-C.; Chen, J.-M.; Hu, Z.; Ni, M.; Shao, Z. Identifying a Universal Activity Descriptor and a Unifying Mechanism Concept on Perovskite Oxides for Green Hydrogen Production. *Adv. Mater.* **2023**, *35* (44), 2305074.

(3) Xu, H.; Guan, D. Exceptional Anisotropic Noncovalent Interactions in Ultrathin Nanorods: The Terminal σ-Hole. *ACS Appl. Mater. Interfaces* **2022**, *14* (45), 51190–51199.

(4) Xiao, W.; Yoo, K.; Kim, J.-H.; Xu, H. Breaking Barriers to High-Practical Li-S Batteries with Isotropic Binary Sulfiphilic Electrocatalyst: Creating a Virtuous Cycle for Favorable Polysulfides Redox Environments. *Adv. Sci.* **2023**, *10* (33), 2303916.

(5) Yang, Y.; Sun, M.; Chen, Z.; Xu, H.; Wang, X.; Duan, J.; Hou, B. 3D Nanothorn Cluster-like Zn-Bi2S3 Sensitized WO_3/ZnO Multijunction with Electron-Storage Characteristic and Adjustable Energy Band for Improving Sustained Photoinduced Cathodic Protection Application. *Chem. Eng. J.* **2023**, *458*, 141458.

(6) Martínez, L.; Andrade, R.; Birgin, E. G.; Martínez, J. M. PACKMOL: A package for building initial configurations for molecular dynamics simulations. *J. Comput. Chem.* **2009**, *30* (13), 2157–2164.

(7) Jorgensen, W. L.; Maxwell, D. S.; Tirado-Rives, J. Development and Testing of the OPLS All-Atom Force Field on Conformational Energetics and Properties of Organic Liquids. *J. Am. Chem. Soc.* **1996**, *118*, 11225-11236.
